# Clinical Impact of Switching or Continuation of Apixaban or Rivaroxaban among Patients with Non-Valvular Atrial Fibrillation

**DOI:** 10.3390/jcm13041073

**Published:** 2024-02-14

**Authors:** Steven Deitelzweig, Amiee Kang, Jenny Jiang, Chuan Gao, Xuemei Luo, Nipun Atreja, Stella Han, Dong Cheng, Saarusri R Loganathan, Gregory Y. H. Lip

**Affiliations:** 1 Ochsner Clinic Foundation, New Orleans, LA 70121, USA; 2 Bristol Myers Squibb, Lawrenceville, NJ 08648, USA; amiee.kang@bms.com (A.K.); jenny.jiang@bms.com (J.J.); chuan.gao@bms.com (C.G.); nipun.atreja@bms.com (N.A.); stella.han@bms.com (S.H.); dong.cheng@bms.com (D.C.); 3 Pfizer Inc., Groton, CT 06340, USA; xuemei.luo@pfizer.com; 4 Mu Sigma Business Solutions Pvt. Ltd., Bangalore 560066, India; saarusri.rl@mu-sigma.com; 5 Liverpool Centre for Cardiovascular Science, University of Liverpool, Liverpool John Moores University and Liverpool Heart & Chest Hospital, Liverpool L14 3PE, UK; 6 Danish Center for Health Services Research, Department of Clinical Medicine, Aalborg University, 9220 Aalborg, Denmark

**Keywords:** direct oral anticoagulants, non-valvular atrial fibrillation, real world, stroke, major bleeding, switching

## Abstract

Background: Real-world evidence on direct oral anticoagulant outcomes among Non-Valvular Atrial Fibrillation (NVAF) patients is limited. We aimed to evaluate stroke/systemic embolism (SE) and major bleeding (MB) risks among NVAF patients continuing or switching to different oral anticoagulants. Methods: Using Optum’s de-identified Clinformatics® Data Mart Database, we identified NVAF patients initiating apixaban or rivaroxaban between 1 January 2013 and 31 December 2021. Patients switching therapies within 30 days before or 90 days after discontinuing their initial DOAC and those who continued initial therapy were included. The index date was the switch date for switchers, while continuers were assigned a hypothetic index date. Switchers and continuers were propensity score matched based on pre-index characteristics. Results: Among 167,868 apixaban and 65,888 rivaroxaban initiators, 2900 apixaban-to-rivaroxaban switchers were matched with 14,500 apixaban continuers, and 2873 rivaroxaban-to-apixaban switchers were matched with 14,365 rivaroxaban continuers. Apixaban-to-rivaroxaban switching was associated with higher stroke/SE risk (HR: 1.99, 95% CI: 1.38–2.88) and MB risk (HR:1.80, 95% CI: 1.46–2.23) than continuing apixaban. Rivaroxaban-to-apixaban switching had similar stroke/SE risk (HR: 0.74, 95% CI: 0.45–1.22) but lower MB risk (HR: 0.49, 95% CI: 0.38–0.65) than continuing rivaroxaban. Conclusions: These findings may aid physicians and patients in making informed decisions when considering a switch between apixaban and rivaroxaban.

## 1. Introduction

Atrial fibrillation (AF) is a medical condition characterised by chaotic and irregular electrical activity in the upper chambers of the heart. It is the most common heart dysrhythmia diagnosed in the United States (US) [1,2]. The incidence of AF is increasing alongside population ageing, and this trend is expected to continue in the coming decades, with AF expected to affect 6–12 million people in the US by 2050 [3]. AF is associated with a substantial economic burden and increased healthcare resource utilisation due to both AF itself and associated complications [4,5].

Non-valvular AF (NVAF)—AF without rheumatic valvular disease is the most common type of AF and a strong independent predictor of stroke [6,7]. Vitamin K antagonists, such as warfarin, were the standard therapy for stroke prevention in NVAF patients until recently. Direct oral anticoagulants (DOACs) such as apixaban, dabigatran, edoxaban, and rivaroxaban are now the standard of care for stroke prevention in NVAF patients in the US [8,9,10,11,12,13]. DOACs are more convenient for patients and have similar or better efficacy and safety than warfarin [8,14].

Switching between DOACs happens frequently in clinical practice [15]. Patients may switch from one DOAC to another for various reasons, such as a lack of effectiveness and side effects of the medication [16,17,18]. In addition to medical reasons, patients may also switch DOAC due to formulary restrictions such as formulary exclusion or increased out-of-pocket costs associated with formulary tier increase [19]. Though prior studies compared the effectiveness and safety of DOACs when used as initial treatments, there are limited data comparing the clinical outcomes between patients who stay on their initial DOAC versus those who switch to a different DOAC in a real-world setting. This retrospective real-world study was performed to try to fill this data gap. Specifically, this study evaluated the risks of stroke/systemic embolism (SE) and major bleeding (MB) in NVAF patients who initiated and continued apixaban use versus those who initiated apixaban use but switched to rivaroxaban, as well as in those who initiated and continued on rivaroxaban versus those who initiated rivaroxaban but switched to apixaban. Apixaban and rivaroxaban are the most commonly used DOACs; therefore, they were the only DOACs considered in this study.

## 2. Materials and Methods

### 2.1. Data Source

This study used de-identified data from Optum’s de-identified Clinformatics^®^ Data Mart Database, which contains data from around 75 million privately insured individuals covered by one of the largest insurance providers in the US from January 2007 to June 2022. The database includes medical and pharmacy claims data for individuals and dependents with commercial employer-sponsored insurance and individuals with Medicare insurance. For this retrospective observational study, data were extracted for the period from 1 January 2012 to 30 June 2022 (the study period).

### 2.2. Patient Selection

The study population included adult (≥18 years) AF patients who initiated apixaban or rivaroxaban between 01 January 2013 and 31 December 2021 (study identification period). The first DOAC prescription date was designated as the DOAC initiation date. The reason that this study only included apixaban and rivaroxaban initiators is due to the following: (1) a separate study assessed patients who initiated warfarin and later switched to other DOACs; (2) sample size for patients who initiated dabigatran or edoxaban and later switched to other DOACs is too low. Patients were included if they had at least one claim with an AF diagnosis code (International Classification of Diseases, 10th Revision [ICD-10] I48.0, I48.1, I48.2, I48.91; ICD, 9th Revision [ICD-9] 42731) prior to or on DOAC initiation date. Included patients also had to have at least 6 months of continuous enrolment before and on their DOAC initiation date. Patients with valvular heart disease, heart valve replacement/transplant, venous thromboembolism, or transient AF (pericarditis, hyperthyroidism, thyrotoxicity) in the 6 months prior to or on the DOAC initiation date were excluded, as were patients with hip or knee replacement surgery 6 weeks prior to or on the DOAC initiation date, patients who were pregnant at any time during the study period, and patients with no follow-up information. Patients with any oral anticoagulant (OAC) prescription in the 6 months before DOAC initiation were also excluded, as were patients with >1 OAC prescription claim on the DOAC initiation date.

Within the apixaban and rivaroxaban initiators, patients were divided into two groups for analysis: switchers and continuers. The switcher group was defined as patients with at least one claim for a different DOAC within 30 days before discontinuing their initial DOAC to 90 days after discontinuing their initial DOAC. Among switchers whose DOAC switch occurred within 30 days before discontinuing their initial DOAC, patients were further excluded if they had stroke/SE or MB event during the period from DOAC switch to the discontinuation of the initiation drug. The reason for this additional exclusion is that it is impossible to determine whether the stroke/SE or MB event was associated with the initial DOAC or the DOAC that the patient switched to due to the overlap of both drugs during the short period. The index date was the date that DOAC switch occurred for the switcher group. Patients were followed from the day after the index date until the earliest of an outcome of interest, discontinuation of the DOAC that was switched to, switch to another OAC, the end of the study period, the end of continuous enrolment, or death.

The continuer group was defined as patients with at least 2 prescription claims for the initial DOAC and no evidence of switching as defined above. In the continuer group, a pseudo-switch date was randomly assigned. A list of pseudo-switch dates were generated based on the distribution of the time from initial DOAC prescription date to the switch date from the switcher cohort. The pseudo-switch date served as the index date for the continuer group. Continuers were followed from the day after the index date until an outcome of interest, DOAC discontinuation, the end of the study period, the end of continuous enrolment, or death, whichever occurred earlier.

Across the analyses, discontinuation was defined as no evidence of the DOAC of interest for 30 days from the last supplied day of the last filled prescription. However, for the continuer group, if patients reinitiated the initial DOAC within 90 days from the last day of the last filled prescription, the date of discontinuation for these patients was defined as the end of treatment after re-initiation of the initial DOAC. Initiators of apixaban or rivaroxaban who discontinued after a single filled prescription were excluded from the analysis.

### 2.3. Study Measures

Baseline demographic and clinical characteristics were calculated for all patients, including age, gender, and comorbidities, as well as stroke/SE and MB events occurring between DOAC initiation and the index date. A complete list of study variables can be found in Table 1 and Table S1. The study outcomes were stroke/SE and MB events during the follow-up period. Stroke/SE events included ischemic stroke, haemorrhagic stroke, and SE. MB events included gastrointestinal bleeding, intracranial haemorrhage, and MB at other key sites. ICD-10 codes used to identify stroke/SE and MB events can be found in Supplemental Table S2. Stroke/SE and MB events were retrospectively identified based on hospitalization claims, with stroke/SE and MB as the first listed diagnosis.

### 2.4. Statistical Analysis

Baseline demographic and clinical characteristics were described for the switcher and continuer groups among the apixaban and rivaroxaban initiators (frequencies and percentages for categorical variables; mean, standard deviation [SD], median, interquartile range [IQR], minimum and maximum for continuous variables). The characteristics were compared using appropriate statistical tests, such as a *t*-test for continuous variables and a chi-squared or Fisher exact test for dichotomous/categorical variables. To ensure the switcher and continuer groups were comparable, they were propensity score matched (PSM) based on their baseline characteristics, duration from their initial DOAC prescription to the index date, and MB and stroke events between DOAC initiation and index date with a ratio of 1:5. The PSM used nearest neighbour matching method without replacement and with a calliper of 0.01. The balance of time duration from initiation to index, baseline demographic, and clinical characteristics was checked based on standardized differences with a threshold of 10%.

For the study outcomes, the event rates for stroke/SE and MB were calculated as per 100 person-years for the PSM switcher and continuer groups among the apixaban and rivaroxaban initiators. The risks of stroke/SE and MB were compared between the PSM switcher and continuer groups, specifically, apixaban-to-rivaroxaban switchers vs. apixaban continuers and rivaroxaban-to-apixaban switchers vs. rivaroxaban continuers using a Cox proportional hazards model. MB and Stroke events prior to the index date for the rivaroxaban-to-apixaban cohort had higher standardized difference and, hence, were included in the Cox model for further adjustment.

A sensitivity analysis was conducted by selecting the patients who were initiated with standard dose of apixaban and rivaroxaban. Same analyses were performed on this selected patient cohort.

## 3. Results

### 3.1. Study Population

**Figure 1:**
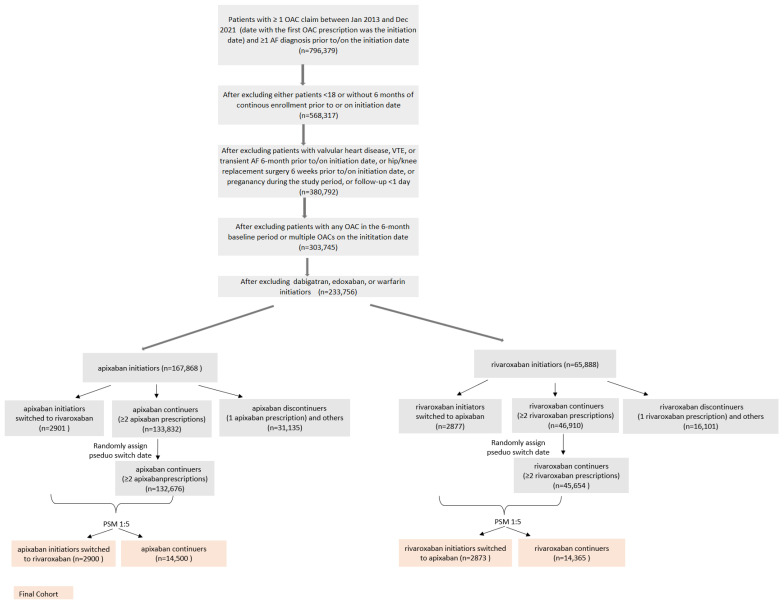
Patient selection criteria. Abbreviations: AF, atrial fibrillation; OAC, oral anticoagulants; PSM, propensity score matching; VTE, venous thromboembolism.

The patient dispositions are shown in Figure 1. After applying inclusion and exclusion criteria, there were 167,868 apixaban initiators, of whom 2901 patients were apixaban-to-rivaroxaban switchers and 133,832 patients were apixaban continuers; 31,135 did not qualify for either group. There were 65,888 rivaroxaban initiators, of whom 2877 were rivaroxaban-to-apixaban switchers and 46,910 were rivaroxaban continuers; 16,101 did not qualify for either group. Before PSM, 2901 patients were included in the apixaban-to-rivaroxaban switchers group, 132,676 patients in the apixaban continuers group, 2877 patients in the rivaroxaban-to-apixaban switchers group, and 45,654 patients in the rivaroxaban continuers group (Table S1).

### 3.2. Patient Characteristics before Propensity Score Matching

Table S1 shows patient characteristics for apixaban-to-rivaroxaban switchers vs. apixaban continuers among apixaban initiators and rivaroxaban-to-apixaban switchers vs. rivaroxaban continuers among rivaroxaban initiators before PSM. Patient characteristics were similar between the apixaban-to-rivaroxaban switchers and apixaban continuers prior to PSM (STD < 10 for most characteristics). However, there were big differences in patient characteristics between rivaroxaban-to-apixaban switchers and rivaroxaban continuers. Compared to rivaroxaban continuers, rivaroxaban-to-apixaban switchers were older (74 vs. 72 years), more likely to be female (48% vs. 41%), had higher mean CCI score (2.86 vs. 2.16), higher mean CHA_2_DS_2_-VASC score (4.06 vs. 3.51), higher mean HAS-BLED score (3.00 vs. 2.49), higher chance to have various comorbidities, and more likely to have stroke/SE and MB events between the rivaroxaban initiation and index dates (Table S1).

### 3.3. Patient Characteristics after PSM

After PSM, the 2900 patients who switched from apixaban to rivaroxaban were matched to 14,500 patients who continued apixaban, and the 2873 patients who switched from rivaroxaban to apixaban were matched to 14,365 patients who continued rivaroxaban (Table 1). Following PSM, all patient characteristics were balanced between the apixaban to rivaroxaban switchers and apixaban continuers. While most of the patient characteristics were balanced between rivaroxaban-to-apixaban switchers and rivaroxaban continuers after the PSM, rivaroxaban-to-apixaban switchers were still more likely to have stroke/SE and MB events between the rivaroxaban initiation and index date than rivaroxaban continuers (STD > 10). The standard dosages of apixaban and rivaroxaban were most commonly prescribed at initiation across the switcher and continuer cohorts (74–84% across cohorts) (Table 1).

### 3.4. Study Outcomes

**Figure 2:**
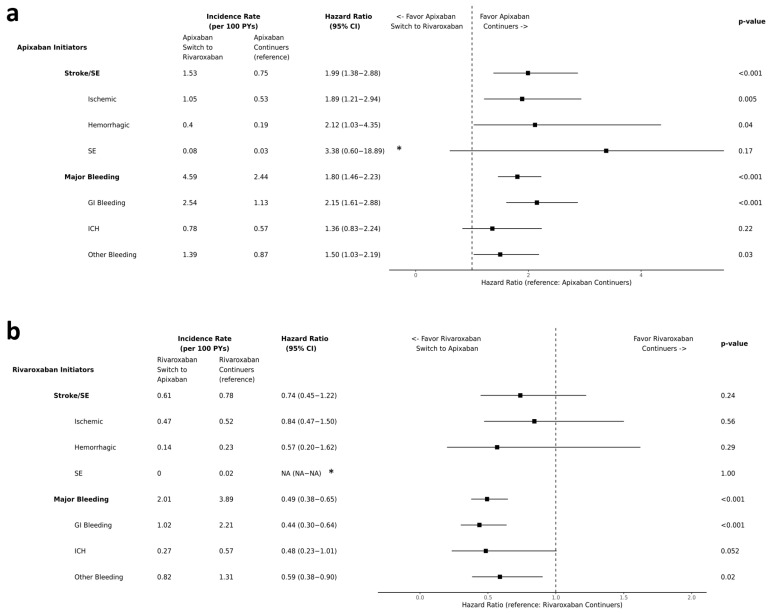
(**a**) Comparison of risks of stroke/SE and MB between apixaban-to-rivaroxaban switchers and apixaban continuers among apixaban initiators. * CI for SE was truncated (**b**) Comparison of risks of stroke/SE and MB between rivaroxaban-to-apixaban switchers and rivaroxaban continuers among rivaroxaban initiators. * Proper Hazard ratio and CI were not obtained due to one group having 0 event. Abbreviation: SE—systemic embolism; GI—gastrointestinal; ICH—intracranial hemorrhage; MB: major bleeding; CI—confidence interval; PYs: person-years.

After PSM, the mean follow-up duration was similar across the switcher and continuer cohorts (314–391 days) (Table 1). Among the apixaban initiators, the incidence rate of stroke/SE and MB was, respectively, 1.53 and 4.59 for apixaban-to-rivaroxaban switchers and 0.75 and 2.44 for apixaban continuers (Figure 2a).

Compared to apixaban continuers, patients switching from apixaban to rivaroxaban were associated with a higher risk of stroke/SE (HR [95% CI]:1.99 [1.38–2.88]) and MB (1.80, [1.46–2.23]). When evaluating individual components of stroke/SE and MB, the apixaban-to-rivaroxaban switchers were also associated with a higher risk of ischemic stroke (1.89 [1.21–2.94]), higher risk of haemorrhagic stroke (2.12 [1.03–4.35], higher risk of GI bleeding (2.15 [1.61–2.88]), and higher risk of other bleeding (1.50 [1.03–2.19]) compared to apixaban continuers (Figure 2a).

Among the rivaroxaban initiators, the incidence rate of stroke/SE and MB was 0.61 and 2.01 for the rivaroxaban-to-apixaban switchers and 0.78 and 3.89 for the rivaroxaban continuers (Figure 2b).

Compared to rivaroxaban continuers, patients switching from rivaroxaban to apixaban were associated with a similar risk of stroke/SE (0.74 [0.45–1.22]) and a lower risk of MB (0.49 [0.38–0.65]) as well as lower risk of GI bleeding (0.44 [0.30–0.64]) and lower risk of other bleeding (0.59 [0.38–0.90]) (Figure 2b).

### 3.5. Sensitivity Analyses

The findings of the sensitivity analysis were consistent with that of the main analysis. Among patients who initiated the standard dose of apixaban, the apixaban-to-rivaroxaban switchers were associated with a higher risk of stroke/SE (1.69 [1.03–2.76]) and MB (1.76 [1.36–2.29]) than apixaban continuers (Figure S1a).

Among patients who initiated the standard dose of rivaroxaban, rivaroxaban-to-apixaban switchers were associated with a similar risk of stroke/SE (0.76 [0.42–1.39]) and a lower risk of MB (0.61 [0.45–0.82]) compared to rivaroxaban continuers (Figure S1b).

## 4. Discussion

This retrospective study was conducted to compare stroke/SE and MB between NVAF patients who initiated and continued the use of a specific DOAC (apixaban or rivaroxaban) and those who switched from their initial DOAC to the other DOAC in routine clinical practice in the US. The study found that switching from apixaban to rivaroxaban was associated with a higher risk of stroke/SE and MB vs. continuous apixaban treatment, while switching from rivaroxaban to apixaban was associated with a similar risk of stroke/SE and a lower risk of MB vs. continuous rivaroxaban treatment. As the American College of Cardiology, American Heart Association, Heart Rhythm Society, and European Society of Cardiology all recommend DOACs instead of warfarin to reduce the risk of stroke/SE for patients with atrial fibrillation, the current study adds to the body of evidence regarding the differential risks of stroke/SE and MB in NVAF patients receiving different DOACs [20,21].

It is not clear what the reasons are for patients switching from apixaban to rivaroxaban or from rivaroxaban to apixaban. Patients may switch treatment for medical or non-medical reasons. A comparison of pre-PSM patient characteristics between patients who switched from apixaban to rivaroxaban and patients who continued the use of apixaban shows that the two groups had very similar demographic and clinical characteristics. The occurrence of stroke/SE and MB events before the index date and baseline all-cause hospitalization were also similar between the two groups. These findings suggest that the switches from apixaban to rivaroxaban were not likely to be caused by medical reasons. Whether the switches were caused by non-medical reasons, such as formulary restrictions, requires further investigation. In contrast, big differences in patient characteristics were found between patients who switched from rivaroxaban to apixaban and patients who continued rivaroxaban. The rivaroxaban-to-apixaban switchers had a higher mean CCI, higher mean CHA_2_DS_2_-VASC, higher mean HAS-BLED, higher chance to have various comorbidities, and were more likely to experience stroke/SE and MB events before the index date than the rivaroxaban continuers. The higher stroke and bleeding risk and the higher chance of having stroke/SE and MB events before the index date among rivaroxaban-to-apixaban switchers suggest that the switch from rivaroxaban to apixaban may be due to medical reasons. Whether other reasons also contribute to the switching warrants a future investigation.

Different outcomes were found to be associated with different types of switches. In this study, the switch from apixaban to rivaroxaban was associated with a higher risk of stroke/SE and MB compared to continuous apixaban treatment. In contrast, the switch from rivaroxaban to apixaban was associated with a similar risk of stroke/SE and a lower risk of MB compared to continuous rivaroxaban treatment. These findings demonstrate that not all DOAC switches are the same. The outcomes depend on which DOAC the patient switched to using. Switching from apixaban to rivaroxaban, especially for non-medical reasons, may have negative consequences.

The findings of this study are in agreement with the body of observational studies that demonstrated a lower or similar risk of stroke/SE and a lower risk of MB for apixaban compared to rivaroxaban in patients with NVAF [22,23,24]. It is important to note that the previous observational studies are somewhat different from the current study. The previous studies compared NVAF patients who initiated different DOACs. Patients were generally censored when the use of the initial DOAC was switched to the use of a different medication, and outcomes after the switch from the initial DOAC were not assessed. The current study includes NVAF patients who initiated the use of a DOAC and then switched to a different DOAC, and the outcomes associated with the DOAC switch were evaluated.

This study has some limitations. Similar to other retrospective observational studies, only a statistical association rather than a causal relationship can be determined between the exposures and outcomes of interest. Although continuer and switcher groups were matched through PSM, potential residual confounders could exist. Renal dysfunction is often one of the drivers for the switch to apixaban. However, serum creatinine clearance and body weight data were not available in the dataset for us to evaluate dose reduction criteria for apixaban and rivaroxaban. The fact that the claims database used in the current study lacked information on lab values, such as serum creatinine clearance, prevented us from evaluating renal function. The severity and stages of renal dysfunction may not be completely balanced between switches and continuers. Medications were based on pharmacy fills, and we were unable to determine if a patient took their medication as prescribed. The reasons for switching from apixaban to rivaroxaban or from rivaroxaban to apixaban could not determined. The sample size for the switcher group is much smaller than the sample size for the continuers. The findings of this study cannot be generalised to all NVAF in the United States since patients with Medicare fee-for-service plans, other public insurance plans, or those who were uninsured were not included.

## 5. Conclusions

Switching from apixaban to rivaroxaban was associated with a higher risk of stroke/SE and MB vs. continuous apixaban treatment, while switching from rivaroxaban to apixaban was associated with a similar risk of stroke/SE and a lower risk of MB vs. continuous rivaroxaban treatment. A sensitivity analysis using the standard dose revealed a consistent pattern in the overall findings. These findings may aid physicians and patients in making informed decisions when considering a switch between apixaban or rivaroxaban. Subsequent studies should be conducted to investigate the reasons for the DOAC switching among NVAF patients.

## Figures and Tables

**Table 1:** Patient characteristics of apixaban and rivaroxaban initiators between switchers and continuers after propensity score matching.

	Apixaban Initiators					Rivaroxaban Initiators				
	Apixaban-to-Rivaroxaban Switchers		Apixaban Continuers		STD ^#^	Rivaroxaban-to-Apixaban Switchers		Rivaroxaban Continuers		STD ^#^
	n/Mean %/SD		n/Mean %/SD			n/Mean %/SD		n/Mean %/SD		
**Sample Size**	2900	100%	14,500	100%		2873	100%	14,365	100%	
**Age**	74.8	9.4	75.0	9.5	1.7	74.1	9.9	74.1	9.8	0.3
Age Group *										
18–54	92	3.2%	412	2.8%	0.0	111	3.9%	543	3.8%	5.1
55–64	286	9.9%	1451	10.0%		331	11.5%	1551	10.8%	
65–74	917	31.6%	4631	31.9%		899	31.3%	4732	32.9%	
75–79	595	20.5%	3034	20.9%		619	21.6%	3067	21.4%	
≥80	1010	34.8%	4972	34.3%		913	31.8%	4472	31.1%	
Gender *^(1)^										
Female	1475	50.9%	7418	51.2%	0.0	1375	47.9%	6968	48.5%	1.3
Male	1424	49.1%	7078	48.8%		1498	52.1%	7397	51.5%	
**Comorbidity Scores**										
CCI score *	2.7	2.5	2.6	2.5	4.7	2.9	2.6	2.8	2.5	2.9
CHA_2_DS_2_-VASC Score	4.1	1.7	4.0	1.7	2.9	4.1	1.8	4.0	1.8	2.4
0	36	1.2%	187	1.3%	3.6	41	1.4%	204	1.4%	3.1
1	137	4.7%	732	5.1%		154	5.4%	787	5.5%	
2	355	12.2%	1916	13.2%		353	12.3%	1867	13.0%	
3	602	20.8%	2956	20.4%		579	20.2%	2819	19.6%	
≥4	1770	61.0%	8709	60.1%		1746	60.8%	8688	60.5%	
HAS-BLED Score ^(2)^	2.9	1.3	2.8	1.3	2.8	3.0	1.3	3.0	1.3	4.0
0	55	1.9%	281	1.9%	2.3	54	1.9%	260	1.8%	8.8
1	339	11.7%	1810	12.5%		295	10.3%	1652	11.5%	
2	851	29.3%	4330	29.9%		727	25.3%	3841	26.7%	
≥3	1655	57.1%	8079	55.7%		1797	62.6%	8612	60.0%	
**Baseline Comorbidities**										
Any bleeding History *	453	15.6%	2257	15.6%	0.2	811	28.2%	3986	27.8%	1.1
Congestive heart failure *	831	28.7%	4020	27.7%	2.1	977	34.0%	4814	33.5%	1.1
Diabetes *	941	32.5%	4500	31.0%	3.0	950	33.1%	4653	32.4%	1.4
Hypertension *	2477	85.4%	12,327	85.0%	1.1	2501	87.1%	12,497	87.0%	0.2
Renal disease *	786	27.1%	3838	26.5%	1.4	898	31.3%	4441	30.9%	0.7
Liver disease *	166	5.7%	760	5.2%	2.1	185	6.4%	875	6.1%	1.4
Myocardial infarction *	335	11.6%	1610	11.1%	1.4	362	12.6%	1743	12.1%	1.4
Dyspepsia or Stomach discomfort *	402	13.9%	1940	13.4%	1.4	346	12.0%	1688	11.8%	0.9
Peripheral vascular disease *	766	26.4%	3589	24.8%	3.8	729	25.4%	3622	25.2%	0.4
Transient ischemic attack *	410	14.1%	1962	13.5%	1.8	370	12.9%	1680	11.7%	3.6
Alcoholism *	87	3.0%	415	2.9%	0.8	77	2.7%	360	2.5%	1.1
Peripheral arterial disease *	408	14.1%	1864	12.9%	3.6	380	13.2%	1893	13.2%	0.1
Coronary artery disease *	1157	39.9%	5643	38.9%	2.0	1182	41.1%	5774	40.2%	1.9
Stroke/SE *	353	12.2%	1671	11.5%	2.0	313	10.9%	1352	9.4%	4.9
All-cause Hospitalization	1118	38.6%	5088	35.1%	7.2	1195	41.6%	4989	34.7%	14.2
Time to Switch *	145.0	203.4	138.9	187.4	3.1	221.5	321.1	213.9	291.5	2.5
Event after DOAC initiation before index date *										
Stroke/SE event after OAC initiation before index date *	44	1.5%	221	1.5%	0.1	66	2.3%	103	0.7%	13
MB event after OAC initiation before index date *	32	1.1%	156	1.1%	0.3	155	5.4%	440	3.1%	11.6
Dosage **										
Low dose	463	16.0%	2627	18.1%	25.8	679	23.6%	3356	23.4%	20.2
Standard dose	2437	84.0%	11,873	81.9%		2131	74.2%	10,770	75.0%	
Other	0	0%	0	0.0%		63	2.2%	239	1.7%	
**Follow-up duration in days** ^(3)^	313.9	348.2	390.9	415.0	20.1	377.9	445.5	355.3	427.5	5.2

Abbreviations: CCI, Charlson Comorbidity Index; CHA_2_DS_2_-VASC, Congestive heart failure, Hypertension, Age ≥ 75 years, Diabetes, Stroke, Vascular disease, Age 65–74 years, Sex; DOAC, Direct Oral Anticoagulants; HAS-BLED, Hypertension, Abnormal renal/liver function, Stroke, Bleeding history or predisposition, Labile international normalized ratio, Elderly, Drugs/alcohol; MB, Major Bleeding; OAC, Oral Anticoagulants; SD, Standard Deviation; SE, Systemic Embolism; STD, Standardized mean Difference. ^#^ STD Difference = 100 × |actual STD diff|. STD Difference greater than 10 is considered significant. * Variables used for PS matching. ^(1)^ Five patients with unknown gender were observed from apixaban initiators. ^(2)^ Because the international normalized ratio value was not available in the database, a modified HAS-BLED score was calculated with a range of 0 to 8. ^(3)^ Follow-up duration is defined from the day after the index date to the earliest of treatment end, death, enrolment end, or study end. ** Apixaban low dose: 2.5 mg, apixaban standard dose 5 mg; rivaroxaban low dose 10 mg, 15 mg, rivaroxaban standard dose 20 mg, the dosage is considered as of the initiation DOAC.

## Data Availability

Restrictions apply to the availability of these data. The datasets generated and/or analyzed for the current study are not publicly available due to the confidential and proprietary nature of the datasets.

## Supplementary Materials

The following supporting information can be downloaded at: https://www.mdpi.com/article/10.3390/jcm13041073/s1, Table S1: Patient characteristics of apixaban and rivaroxaban initiators between switchers and continuers before propensity score matching; Table S2: Study codes used to identify MB and Stroke; Figure S1a: Comparison of risks of stroke/SE and MB between apixaban-to-rivaroxaban switchers and apixaban continuers among apixaban initiators—standard dose; Figure S1b: Comparison of risks of stroke/SE and MB between rivaroxaban-to-apixaban switchers and rivaroxaban continuers among rivaroxaban initiators—standard dose.
