# Supplementary material for: Clinical Impact of Switching or Continuation of Apixaban or Rivaroxaban among Patients with Non-Valvular Atrial Fibrillation
Source: J Clin Med. 2024 Feb 14;13(4):1073. doi: 10.3390/jcm13041073 (PMC10889502; doi:10.3390/jcm13041073)
Supplement: Supplementary file 1 [file jcm-13-01073-s001.zip › jcm-2783197-supplementary.pdf]

Supplementary table

Table S1 Patient characteristics of Apixaban and Rivaroxaban initiators between switchers and continuers before Propensity Score Matching

	Apixaban initiators					Rivaroxaban initiators				
	Apixaban to rivaroxaban switchers		Apixaban continuers		STD #	Rivaroxaban to apixaban switchers		Rivaroxaban continuers		STD #
	n/Mean	%/SD	n/Mean	%/SD		n/Mean	%/SD	n/Mean	%/SD	
**Sample Size**	2,901	100%	132,676	100%		2,877	100%	45,654	100%	
**Age**	74.8	9.4	74.8	9.9	0.3	74.1	9.9	72.2	10.8	18.5
**Age Group***										
18—54	92	3.2%	4,737	3.6%		111	3.9%	2,997	6.6%	
55—64	286	9.9%	13,682	10.3%		331	11.5%	6,835	15.0%	
65—74	918	31.6%	42,314	31.9%	7.2	899	31.3%	15,432	33.8%	21.1
75—79	595	20.5%	25,153	19.0%		619	21.5%	7,817	17.1%	
≥80	1,010	34.8%	46,790	35.3%		917	31.9%	12,573	27.5%	
**Gender*(1)**										
Female	1,475	50.8%	62,721	47.3%	8.0	1,377	47.9%	18,682	40.9%	14.1
Male	1,425	49.1%	69,940	52.7%		1,500	52.1%	26,967	59.1%	
**Comorbidity Scores**										
CCI Score*	2.7	2.4	2.8	2.5	2.5	2.9	2.6	2.2	2.2	29.1
CHA_2DS_2-VASC Score	4.1	1.7	4.0	1.7	2.2	4.1	1.8	3.5	1.7	31.2
0	36	1.2%	1,976	1.5%		41	1.4%	1,420	3.1%	
1	137	4.7%	6,967	5.3%		154	5.4%	4,029	8.8%	
2	356	12.3%	16,736	12.6%		353	12.3%	7,925	17.4%	
3	602	20.8%	26,436	19.9%	3.6	579	20.1%	10,001	21.9%	30.8
≥4	1,770	61.0%	80,561	60.7%		1,750	60.8%	22,279	48.8%	
HAS-BLED Score (2)	2.9	1.3	2.8	1.3	1.6	3.0	1.3	2.5	1.3	39.4
0	55	1.9%	2,803	2.1%	0.0	54	1.9%	1,782	3.9%	38.3
1	339	11.7%	15,744	11.9%		295	10.3%	8,002	17.5%	

	Apixaban initiators					Rivaroxaban initiators				
	Apixaban to rivaroxaban switchers		Apixaban continuers		STD #	Rivaroxaban to apixaban switchers		Rivaroxaban continuers		STD #
	n/Mean	%/SD	n/Mean	%/SD		n/Mean	%/SD	n/Mean	%/SD	
2	851	29.3%	38,766	29.2%		729	25.3%	14,853	32.5%	
≥3	1,656	57.1%	75,363	56.8%		1,799	62.5%	21,017	46.0%	
**Baseline Comorbidities**										
Any bleeding history*	453	15.6%	18,543	14.0%	4.6	813	28.3%	6,148	13.5%	37.0
Congestive heart failure*	831	28.7%	40,009	30.2%	3.3	979	34.0%	10,979	24.1%	22.1
Diabetes*	941	32.4%	46,403	35.0%	5.4	951	33.1%	14,642	32.1%	2.1
Hypertension*	2,478	85.4%	113,496	85.5%	0.4	2,505	87.1%	37,085	81.2%	16.0
Renal disease*	786	27.1%	39,176	29.5%	5.4	900	31.3%	9,104	19.9%	26.2
Liver disease*	166	5.7%	7,134	5.4%	1.5	186	6.5%	1,951	4.3%	9.7
Myocardial infarction*	335	11.6%	15,800	11.9%	1.1	364	12.7%	3,932	8.6%	13.1
Dyspepsia or Stomach discomfort*	402	13.9%	15,768	11.9%	5.9	346	12.0%	3,600	7.9%	13.9
Peripheral vascular disease*	766	26.4%	33,304	25.1%	3.0	730	25.4%	9,288	20.3%	12.0
Transient ischemic attack*	410	14.1%	16,829	12.7%	4.3	370	12.9%	3,749	8.2%	15.2
Alcoholism*	87	3.0%	3,468	2.6%	2.3	77	2.7%	1,108	2.4%	1.6
Peripheral arterial disease*	408	14.1%	16,799	12.7%	4.1	380	13.2%	5,000	11.0%	6.9
Coronary artery disease*	1,158	39.9%	53,736	40.5%	1.2	1,185	41.2%	15,926	34.9%	13.0
Stroke/SE*	353	12.2%	14,352	10.8%	4.2	313	10.9%	3,275	7.2%	13.0
All-cause Hospitalization	1,118	38.5%	47,793	36.0%	5.2	1,197	41.6%	12,078	26.5%	32.4
**Time to switch***	145.7	207.4	136.5	179.1	4.8	224.8	332.6	193.2	259.0	10.6

	Apixaban initiators					Rivaroxaban initiators				
	Apixaban to rivaroxaban switchers		Apixaban continuers		STD #	Rivaroxaban to apixaban switchers		Rivaroxaban continuers		STD #
	n/Mean	%/SD	n/Mean	%/SD		n/Mean	%/SD	n/Mean	%/SD	
**Event after DOAC initiation before index date***										
Stroke/SE event after OAC initiation before index date*	44	1.5%	393	0.3%	12.9	66	2.3%	104	0.2%	18.6
MB event after OAC initiation before index date*	32	1.1%	953	0.7%	4.1	155	5.4%	445	1.0%	25.4
**Dosage****										
Low dose	463	16.0%	24,537	18.5%		679	23.6%	8,806	19.3%	
Standard dose	2,438	84.0%	108,139	81.5%	25.8	2,135	74.2%	36,154	79.2%	22.1
Other	0	0.0%	0	0.0%		63	2.2%	694	1.5%	
**Follow-up duration in days** (3)	313.8	348.2	385.9	410.8	18.9	377.7	445.3	359.4	432.8	4.2

Abbreviations: CCI, Charlson Comorbidity Index; CHA_2DS_2-VASC, Congestive heart failure, Hypertension, Age ≥ 75 years, Diabetes, Stroke, Vascular disease, Age 65–74 years, Sex; DOAC, Direct Oral Anticoagulants; HAS-BLED, Hypertension, Abnormal renal/liver function, Stroke, Bleeding history or predisposition, Labile international normalized ratio, Elderly, Drugs/alcohol; MB, Major Bleeding; OAC, Oral Anticoagulants; SD, Standard Deviation; SE, Systemic Embolism; STD, Standardized mean Difference;

#STD Difference=100 × |actual STD diff|. STD Difference greater than 10 is considered significant.
*Variables used for PS matching.

(1) Sixteen patients with unknown gender were observed from apixaban initiators and five patients with unknown gender were observed from rivaroxaban initiators

(2) Because the international normalized ratio value was not available in the database, a modified HAS-BLED score was calculated with a range of 0 to 8

(3) Follow-up duration is defined from the day after the index date to the earliest of treatment end, death, enrolment end, or study end

**Apixaban low dose: 2.5mg, apixaban standard dose 5mg; rivaroxaban low dose 10mg, 15mg, rivaroxaban standard dose 20mg, the dosage is considered as of the initiation DOAC.

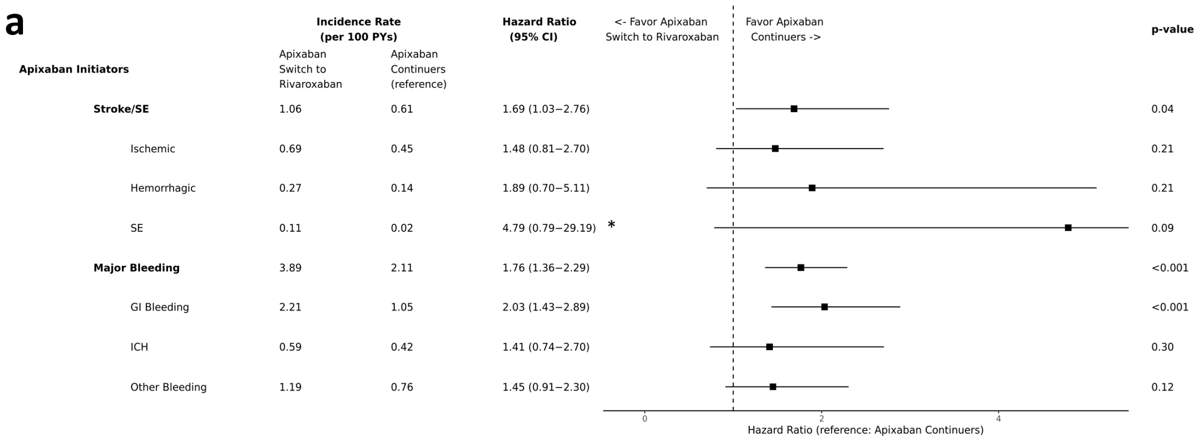

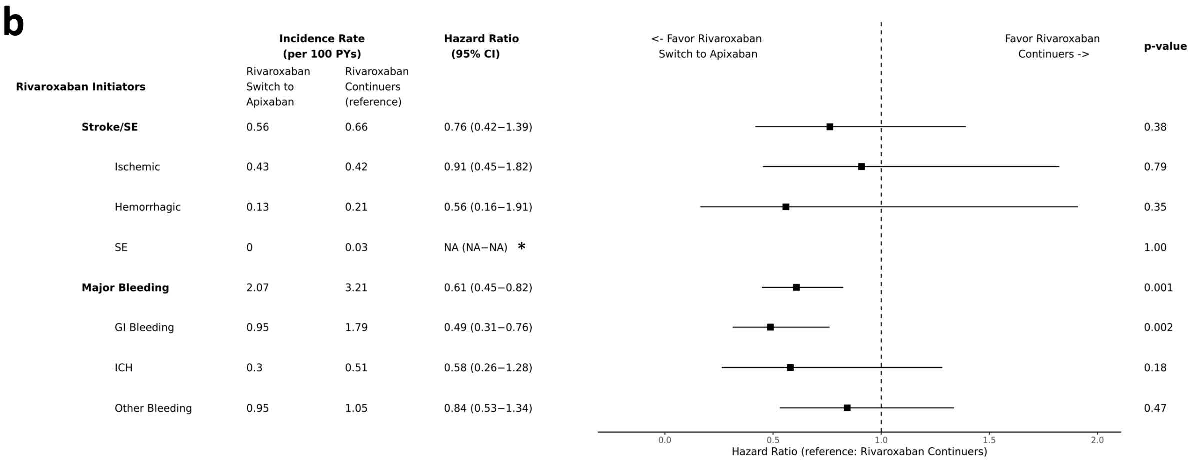

Figure S1. (a) Comparison of risks of stroke/SE and MB between apixaban to rivaroxaban switchers and apixaban continuers among apixaban initiators - standard dose. * CI for SE was truncated **(b)** Comparison of risks of stroke/SE and MB between rivaroxaban to apixaban switchers and rivaroxaban continuers among rivaroxaban initiators - standard dose. * Proper Hazard ratio and CI were not obtained due to one group has 0 event. Abbreviation: SE - systemic embolism; GI - gastrointestinal; ICH - intracranial hemorrhage; MB: major bleeding; CI - confidence interval; PYs: person-years

Table S2 Study codes used to identify MB and Stroke

Code Type	Code	Diagnosis	ICD type	Description
Diagnosis	I6001	Hemorrhagic stroke	ICD10	Nontraumatic Subarachnoid Hemorrhage From Right Carotid Siphon And Bifurcation Or Ntrm Subarach Hemor From Right Carotid Siphon And Bifurc
Diagnosis	I6002	Hemorrhagic stroke	ICD10	Nontraumatic Subarachnoid Hemorrhage From Left Carotid Siphon And Bifurcation Or Ntrm Subarach Hemorrhage From Left Carotid Siphon And Bifurc

Diagnosis	I6010	Hemorrhagic stroke	ICD10	Nontraumatic Subarachnoid Hemorrhage From Unspecified Middle Cerebral Artery Or Ntrm Subarach Hemorrhage From Unsp Middle Cerebral Artery
Diagnosis	I6011	Hemorrhagic stroke	ICD10	Nontraumatic Subarachnoid Hemorrhage From Right Middle Cerebral Artery Or Ntrm Subarach Hemorrhage From Right Middle Cerebral Artery
Diagnosis	I6012	Hemorrhagic stroke	ICD10	Nontraumatic Subarachnoid Hemorrhage From Left Middle Cerebral Artery Or Ntrm Subarach Hemorrhage From Left Middle Cerebral Artery
Diagnosis	I602	Hemorrhagic stroke	ICD10	Nontraumatic Subarachnoid Hemorrhage From Anterior Communicating Artery
Diagnosis	I6030	Hemorrhagic stroke	ICD10	Nontraumatic Subarachnoid Hemorrhage From Unspecified Posterior Communicating Artery Or Ntrm Subarach Hemor From Unsp Posterior Communicating Artery
Diagnosis	I6031	Hemorrhagic stroke	ICD10	Nontraumatic Subarachnoid Hemorrhage From Right Posterior Communicating Artery Or Ntrm Subarach Hemor From Right Post Communicating Artery
Diagnosis	I6032	Hemorrhagic stroke	ICD10	Nontraumatic Subarachnoid Hemorrhage From Left Posterior Communicating Artery Or Ntrm Subarach Hemor From Left Posterior Communicating Artery
Diagnosis	I604	Hemorrhagic stroke	ICD10	Nontraumatic Subarachnoid Hemorrhage From Basilar Artery
Diagnosis	I6050	Hemorrhagic stroke	ICD10	Nontraumatic Subarachnoid Hemorrhage From Unsp Verteb Art Or Nontraumatic Subarachnoid Hemorrhage From Unspecified Vertebral Artery
Diagnosis	I6051	Hemorrhagic stroke	ICD10	Nontraumatic Subarachnoid Hemorrhage From R Verteb Art Or Nontraumatic Subarachnoid Hemorrhage From Right Vertebral Artery
Diagnosis	I6052	Hemorrhagic stroke	ICD10	Nontraumatic Subarachnoid Hemorrhage From L Verteb Art Or Nontraumatic Subarachnoid Hemorrhage From Left Vertebral Artery
Diagnosis	I606	Hemorrhagic stroke	ICD10	Nontraumatic Subarachnoid Hemorrhage From Oth Intracran Art Or Nontraumatic Subarachnoid Hemorrhage From Other Intracranial Arteries
Diagnosis	I607	Hemorrhagic stroke	ICD10	Nontraumatic Subarachnoid Hemorrhage From Unsp Intracran Art Or Nontraumatic Subarachnoid Hemorrhage From Unspecified Intracranial Artery
Diagnosis	I608	Hemorrhagic stroke	ICD10	Other Nontraumatic Subarachnoid Hemorrhage
Diagnosis	I609	Hemorrhagic stroke	ICD10	Nontraumatic Subarachnoid Hemorrhage, Unspecified
Diagnosis	I610	Hemorrhagic stroke	ICD10	Nontraumatic Intcrbl Hemorrhage In Hemisphere, Subcortical Or Nontraumatic Intracerebral Hemorrhage In Hemisphere, Subcortical
Diagnosis	I611	Hemorrhagic stroke	ICD10	Nontraumatic Intcrbl Hemorrhage In Hemisphere, Cortical Or Nontraumatic Intracerebral Hemorrhage In Hemisphere, Cortical

Diagnosis	I612	Hemorrhagic stroke	ICD10	Nontraumatic Intracerebral Hemorrhage In Hemisphere, Unsp Or Nontraumatic Intracerebral Hemorrhage In Hemisphere, Unspecified
Diagnosis	I613	Hemorrhagic stroke	ICD10	Nontraumatic Intracerebral Hemorrhage In Brain Stem
Diagnosis	I614	Hemorrhagic stroke	ICD10	Nontraumatic Intracerebral Hemorrhage In Cerebellum
Diagnosis	I615	Hemorrhagic stroke	ICD10	Nontraumatic Intracerebral Hemorrhage, Intraventricular
Diagnosis	I616	Hemorrhagic stroke	ICD10	Nontraumatic Intracerebral Hemorrhage, Multiple Localized
Diagnosis	I618	Hemorrhagic stroke	ICD10	Other Nontraumatic Intracerebral Hemorrhage
Diagnosis	I619	Hemorrhagic stroke	ICD10	Nontraumatic Intracerebral Hemorrhage, Unspecified
Diagnosis	430	Hemorrhagic stroke	ICD9	Subarachnoid Hemorrhage
Diagnosis	431	Hemorrhagic stroke	ICD9	Intracerebral Hemorrhage
Diagnosis	4320	Hemorrhagic stroke	ICD9	Nontraum Extradural Hem
Diagnosis	4321	Hemorrhagic stroke	ICD9	Subdural Hemorrhage
Diagnosis	4329	Hemorrhagic stroke	ICD9	Intracranial Hemorr Nos
Diagnosis	I6300	Ischemic stroke	ICD10	Cerebral Infarction Due To Thombos Unsp Precerebral Artery Or Cerebral Infarction Due To Thrombosis Of Unspecified Precerebral Artery
Diagnosis	I63011	Ischemic stroke	ICD10	Cerebral Infarction Due To Thrombosis Of R Verteb Art Or Cerebral Infarction Due To Thrombosis Of Right Vertebral Artery
Diagnosis	I63012	Ischemic stroke	ICD10	Cerebral Infarction Due To Thrombosis Of L Verteb Art Or Cerebral Infarction Due To Thrombosis Of Left Vertebral Artery
Diagnosis	I63013	Ischemic stroke	ICD10	Cerebral Infarction Due To Thrombosis Of Bilateral Vertebral Arteries
Diagnosis	I63019	Ischemic stroke	ICD10	Cerebral Infarction Due To Thombos Unsp Vertebral Artery Or Cerebral Infarction Due To Thrombosis Of Unspecified Vertebral Artery
Diagnosis	I6302	Ischemic stroke	ICD10	Cerebral Infarction Due To Thrombosis Of Basilar Artery
Diagnosis	I63031	Ischemic stroke	ICD10	Cerebral Infarction Due To Thrombosis Of Right Carotid Artery Or Cerebral Infrc Due To Thrombosis Of Right Carotid Artery
Diagnosis	I63032	Ischemic stroke	ICD10	Cerebral Infarction Due To Thrombosis Of Left Carotid Artery

Diagnosis	I63033	Ischemic stroke	ICD10	Cerebral Infarction Due To Thrombosis Of Bilateral Carotid Arteries
Diagnosis	I63039	Ischemic stroke	ICD10	Cerebral Infarction Due To Thrombosis Of Unsp Carotid Artery Or Cerebral Infarction Due To Thrombosis Of Unspecified Carotid Artery
Diagnosis	I6309	Ischemic stroke	ICD10	Cerebral Infarction Due To Thrombosis Of Other Precerebral Artery Or Cerebral Infarction Due To Thrombosis Of Precerebral Artery
Diagnosis	I6310	Ischemic stroke	ICD10	Cerebral Infarction Due To Embolism Of Unsp Precerb Artery Or Cerebral Infarction Due To Embolism Of Unspecified Precerebral Artery
Diagnosis	I63111	Ischemic stroke	ICD10	Cerebral Infarction Due To Embolism Of R Verteb Art Or Cerebral Infarction Due To Embolism Of Right Vertebral Artery
Diagnosis	I63112	Ischemic stroke	ICD10	Cerebral Infarction Due To Embolism Of Left Vertebral Artery
Diagnosis	I63113	Ischemic stroke	ICD10	Cerebral Infarction Due To Embolism Of Bilateral Vertebral Arteries
Diagnosis	I63119	Ischemic stroke	ICD10	Cerebral Infarction Due To Embolism Of Unsp Vertebral Artery Or Cerebral Infarction Due To Embolism Of Unspecified Vertebral Artery
Diagnosis	I6312	Ischemic stroke	ICD10	Cerebral Infarction Due To Embolism Of Basilar Artery
Diagnosis	I63131	Ischemic stroke	ICD10	Cerebral Infarction Due To Embolism Of Right Carotid Artery
Diagnosis	I63132	Ischemic stroke	ICD10	Cerebral Infarction Due To Embolism Of Left Carotid Artery
Diagnosis	I63133	Ischemic stroke	ICD10	Cerebral Infarction Due To Embolism Of Bilateral Carotid Arteries
Diagnosis	I63139	Ischemic stroke	ICD10	Cerebral Infarction Due To Embolism Of Unsp Carotid Artery Or Cerebral Infarction Due To Embolism Of Unspecified Carotid Artery
Diagnosis	I6319	Ischemic stroke	ICD10	Cerebral Infarction Due To Embolism Of Other Precerebral Artery Or Cerebral Infarction Due To Embolism Of Precerebral Artery
Diagnosis	I6320	Ischemic stroke	ICD10	Cereb Infrc Due To Unsp Occls Or Stenos Of Unsp Precerb Art Or Cerebral Infarction Due To Unspecified Occlusion Or Stenosis Of Unspecified Precerebral Arteries
Diagnosis	I63211	Ischemic stroke	ICD10	Cereb Infrc Due To Unsp Occls Or Stenos Of Right Verteb Art Or Cerebral Infarction Due To Unspecified Occlusion Or Stenosis Of Right Vertebral Arteries Or Cerebral Infarction Due To Unspecified Occlusion Or Stenosis Of Right Vertebral Artery
Diagnosis	I63212	Ischemic stroke	ICD10	Cereb Infrc Due To Unsp Occls Or Stenosis Of Left Verteb Art Or Cerebral Infarction Due To Unspecified Occlusion Or Stenosis Of Left Vertebral Arteries Or Cerebral Infarction Due To Unspecified Occlusion Or Stenosis Of Left Vertebral Artery

Diagnosis	I63213	Ischemic stroke	ICD10	Cerebral Infarction Due To Unspecified Occlusion Or Stenosis Of Bilateral Vertebral Arteries
Diagnosis	I63219	Ischemic stroke	ICD10	Cereb Infrc Due To Unsp Occls Or Stenosis Of Unsp Verteb Art Or Cerebral Infarction Due To Unspecified Occlusion Or Stenosis Of Unspecified Vertebral Arteries Or Cerebral Infarction Due To Unspecified Occlusion Or Stenosis Of Unspecified Vertebral Artery
Diagnosis	I6322	Ischemic stroke	ICD10	Cerebral Infarction Due To Unspecified Occlusion Or Stenosis Of Basilar Arteries Or Cerebral Infarction Due To Unspecified Occlusion Or Stenosis Of Basilar Artery Or Cerebral Infrc Due To Unsp Occls Or Stenosis Of Basilar Art
Diagnosis	I63231	Ischemic stroke	ICD10	Cereb Infrc Due To Unsp Occls Or Stenos Of Right Carotid Art Or Cerebral Infarction Due To Unspecified Occlusion Or Stenosis Of Right Carotid Arteries
Diagnosis	I63232	Ischemic stroke	ICD10	Cereb Infrc Due To Unsp Occls Or Stenos Of Left Carotid Art Or Cerebral Infarction Due To Unspecified Occlusion Or Stenosis Of Left Carotid Arteries
Diagnosis	I63233	Ischemic stroke	ICD10	Cerebral Infarction Due To Unspecified Occlusion Or Stenosis Of Bilateral Carotid Arteries
Diagnosis	I63239	Ischemic stroke	ICD10	Cereb Infrc Due To Unsp Occls Or Stenos Of Unsp Carotid Art Or Cerebral Infarction Due To Unspecified Occlusion Or Stenosis Of Unspecified Carotid Arteries Or Cerebral Infarction Due To Unspecified Occlusion Or Stenosis Of Unspecified Carotid Artery
Diagnosis	I6329	Ischemic stroke	ICD10	Cerebral Infarction Due To Unspecified Occlusion Or Stenosis Of Other Precerebral Arteries Or Cerebral Infrc Due To Unsp Occls Or Stenosis Of Precerb Art
Diagnosis	I6330	Ischemic stroke	ICD10	Cerebral Infarction Due To Thombos Unsp Cerebral Artery Or Cerebral Infarction Due To Thrombosis Of Unspecified Cerebral Artery
Diagnosis	I63311	Ischemic stroke	ICD10	Cereb Infrc Due To Thombos Of Right Middle Cerebral Artery Or Cerebral Infarction Due To Thrombosis Of Right Middle Cerebral Artery
Diagnosis	I63312	Ischemic stroke	ICD10	Cerebral Infarction Due To Thrombosis Of Left Middle Cerebral Artery Or Cerebral Infrc Due To Thombos Of Left Middle Cerebral Artery
Diagnosis	I63313	Ischemic stroke	ICD10	Cerebral Infarction Due To Thrombosis Of Bilateral Middle Cerebral Arteries
Diagnosis	I63319	Ischemic stroke	ICD10	Cerebral Infarction Due To Thrombosis Of Unspecified Middle Cerebral Artery Or Cerebral Infrc Due To Thombos Unsp Middle Cerebral Artery
Diagnosis	I63321	Ischemic stroke	ICD10	Cerebral Infarction Due To Thrombosis Of Right Anterior Cerebral Artery Or Cerebral Infrc Due To Thombos Of Right Ant Cerebral Artery
Diagnosis	I63322	Ischemic stroke	ICD10	Cerebral Infarction Due To Thrombosis Of Left Anterior Cerebral Artery Or Cerebral Infrc Due To Thombos Of Left Ant Cerebral Artery

Diagnosis	I63323	Ischemic stroke	ICD10	Cerebral Infarction Due To Thrombosis Of Bilateral Anterior Arteries Or Cerebral Infarction Due To Thrombosis Of Bilateral Anterior Cerebral Arteries
Diagnosis	I63329	Ischemic stroke	ICD10	Cerebral Infarction Due To Thrombosis Of Unspecified Anterior Cerebral Artery Or Cerebral Infrc Due To Thombos Unsp Anterior Cerebral Artery
Diagnosis	I63331	Ischemic stroke	ICD10	Cerebral Infarction Due To Thrombosis Of Right Posterior Cerebral Artery Or Cerebral Infrc Due To Thombos Of Right Post Cerebral Artery
Diagnosis	I63332	Ischemic stroke	ICD10	Cerebral Infarction Due To Thrombosis Of Left Posterior Cerebral Artery Or Cerebral Infrc Due To Thombos Of Left Post Cerebral Artery
Diagnosis	I63333	Ischemic stroke	ICD10	Cerebral Infarction Due To Thrombosis Of Bilateral Posterior Cerebral Arteries Or Cerebral Infarction To Thrombosis Of Bilateral Posterior Arteries Or Cerebral Infarction To Thrombosis Of Bilateral Posterior Cerebral Arteries
Diagnosis	I63339	Ischemic stroke	ICD10	Cerebral Infarction Due To Thrombosis Of Unspecified Posterior Cerebral Artery Or Cerebral Infrc Due To Thombos Unsp Posterior Cerebral Artery
Diagnosis	I63341	Ischemic stroke	ICD10	Cerebral Infarction Due To Thrombosis Of Right Cerebellar Artery Or Cerebral Infrc Due To Thrombosis Of Right Cereblr Artery
Diagnosis	I63342	Ischemic stroke	ICD10	Cerebral Infarction Due To Thrombosis Of Left Cerebellar Artery Or Cerebral Infarction Due To Thrombosis Of Left Cereblr Artery
Diagnosis	I63343	Ischemic stroke	ICD10	Cerebral Infarction Due To Thrombosis Of Bilateral Cerebellar Arteries Or Cerebral Infarction To Thrombosis Of Bilateral Cerebellar Arteries
Diagnosis	I63349	Ischemic stroke	ICD10	Cerebral Infarction Due To Thombos Unsp Cerebellar Artery Or Cerebral Infarction Due To Thrombosis Of Unspecified Cerebellar Artery
Diagnosis	I6339	Ischemic stroke	ICD10	Cerebral Infarction Due To Thrombosis Of Oth Cerebral Artery Or Cerebral Infarction Due To Thrombosis Of Other Cerebral Artery
Diagnosis	I6340	Ischemic stroke	ICD10	Cerebral Infarction Due To Embolism Of Unsp Cerebral Artery Or Cerebral Infarction Due To Embolism Of Unspecified Cerebral Artery
Diagnosis	I63411	Ischemic stroke	ICD10	Cereb Infrc Due To Embolism Of Right Middle Cerebral Artery Or Cerebral Infarction Due To Embolism Of Right Middle Cerebral Artery
Diagnosis	I63412	Ischemic stroke	ICD10	Cereb Infrc Due To Embolism Of Left Middle Cerebral Artery Or Cerebral Infarction Due To Embolism Of Left Middle Cerebral Artery
Diagnosis	I63413	Ischemic stroke	ICD10	Cerebral Infarction Due To Embolism Of Bilateral Middle Cerebral Arteries

Diagnosis	I63419	Ischemic stroke	ICD10	Cereb Infrc Due To Embolism Of Unsp Middle Cerebral Artery Or Cerebral Infarction Due To Embolism Of Unspecified Middle Cerebral Artery
Diagnosis	I63421	Ischemic stroke	ICD10	Cerebral Infarction Due To Embolism Of Right Anterior Cerebral Artery Or Cerebral Infrc Due To Embolism Of Right Ant Cerebral Artery
Diagnosis	I63422	Ischemic stroke	ICD10	Cerebral Infarction Due To Embolism Of Left Anterior Cerebral Artery Or Cerebral Infrc Due To Embolism Of Left Ant Cerebral Artery
Diagnosis	I63423	Ischemic stroke	ICD10	Cerebral Infarction Due To Embolism Of Bilateral Anterior Cerebral Arteries
Diagnosis	I63429	Ischemic stroke	ICD10	Cerebral Infarction Due To Embolism Of Unspecified Anterior Cerebral Artery Or Cerebral Infrc Due To Embolism Of Unsp Ant Cerebral Artery
Diagnosis	I63431	Ischemic stroke	ICD10	Cerebral Infarction Due To Embolism Of Right Posterior Cerebral Artery Or Cerebral Infrc Due To Embolism Of Right Post Cerebral Artery
Diagnosis	I63432	Ischemic stroke	ICD10	Cerebral Infarction Due To Embolism Of Left Posterior Cerebral Artery Or Cerebral Infrc Due To Embolism Of Left Post Cerebral Artery
Diagnosis	I63433	Ischemic stroke	ICD10	Cerebral Infarction Due To Embolism Of Bilateral Posterior Cerebral Arteries
Diagnosis	I63439	Ischemic stroke	ICD10	Cerebral Infarction Due To Embolism Of Unspecified Posterior Cerebral Artery Or Cerebral Infrc Due To Embolism Of Unsp Post Cerebral Artery
Diagnosis	I63441	Ischemic stroke	ICD10	Cerebral Infarction Due To Embolism Of Right Cerebellar Artery Or Cerebral Infarction Due To Embolism Of Right Cereblr Artery
Diagnosis	I63442	Ischemic stroke	ICD10	Cerebral Infarction Due To Embolism Of Left Cerebellar Artery Or Cerebral Infarction Due To Embolism Of Left Cereblr Artery
Diagnosis	I63443	Ischemic stroke	ICD10	Cerebral Infarction Due To Embolism Of Bilateral Cerebellar Arteries
Diagnosis	I63449	Ischemic stroke	ICD10	Cerebral Infarction Due To Embolism Of Unsp Cereblr Artery Or Cerebral Infarction Due To Embolism Of Unspecified Cerebellar Artery
Diagnosis	I6349	Ischemic stroke	ICD10	Cerebral Infarction Due To Embolism Of Other Cerebral Artery
Diagnosis	I6350	Ischemic stroke	ICD10	Cereb Infrc Due To Unsp Occls Or Stenos Of Unsp Cereb Artery Or Cerebral Infarction Due To Unspecified Occlusion Or Stenosis Of Unspecified Cerebral Artery
Diagnosis	I63511	Ischemic stroke	ICD10	Cereb Infrc D/T Unsp Occls Or Stenos Of Right Mid Cereb Art Or Cerebral Infarction Due To Unspecified Occlusion Or Stenosis Of Right Middle Cerebral Artery
Diagnosis	I63512	Ischemic stroke	ICD10	Cereb Infrc D/T Unsp Occls Or Stenos Of Left Mid Cereb Art Or Cerebral Infarction Due To Unspecified Occlusion Or Stenosis Of Left Middle Cerebral Artery

Diagnosis	I63513	Ischemic stroke	ICD10	Cerebral Infarction Due To Unspecified Occlusion Or Stenosis Of Bilateral Middle Arteries Or Cerebral Infarction Due To Unspecified Occlusion Or Stenosis Of Bilateral Middle Cerebral Arteries
Diagnosis	I63519	Ischemic stroke	ICD10	Cereb Infrc D/T Unsp Occls Or Stenos Of Unsp Mid Cereb Art Or Cerebral Infarction Due To Unspecified Occlusion Or Stenosis Of Unspecified Middle Cerebral Artery
Diagnosis	I63521	Ischemic stroke	ICD10	Cereb Infrc D/T Unsp Occls Or Stenos Of Right Ant Cereb Art Or Cerebral Infarction Due To Unspecified Occlusion Or Stenosis Of Right Anterior Cerebral Artery
Diagnosis	I63522	Ischemic stroke	ICD10	Cereb Infrc D/T Unsp Occls Or Stenos Of Left Ant Cereb Art Or Cerebral Infarction Due To Unspecified Occlusion Or Stenosis Of Left Anterior Cerebral Artery
Diagnosis	I63523	Ischemic stroke	ICD10	Cerebral Infarction Due To Unspecified Occlusion Or Stenosis Of Bilateral Anterior Arteries Or Cerebral Infarction Due To Unspecified Occlusion Or Stenosis Of Bilateral Anterior Cerebral Arteries
Diagnosis	I63529	Ischemic stroke	ICD10	Cereb Infrc D/T Unsp Occls Or Stenos Of Unsp Ant Cereb Art Or Cerebral Infarction Due To Unspecified Occlusion Or Stenosis Of Unspecified Anterior Cerebral Artery
Diagnosis	I63531	Ischemic stroke	ICD10	Cereb Infrc D/T Unsp Occls Or Stenos Of Right Post Cereb Art Or Cerebral Infarction Due To Unspecified Occlusion Or Stenosis Of Right Posterior Cerebral Artery
Diagnosis	I63532	Ischemic stroke	ICD10	Cereb Infrc D/T Unsp Occls Or Stenos Of Left Post Cereb Art Or Cerebral Infarction Due To Unspecified Occlusion Or Stenosis Of Left Posterior Cerebral Artery
Diagnosis	I63533	Ischemic stroke	ICD10	Cerebral Infarction Due To Unspecified Occlusion Or Stenosis Of Bilateral Posterior Arteries Or Cerebral Infarction Due To Unspecified Occlusion Or Stenosis Of Bilateral Posterior Cerebral Arteries
Diagnosis	I63539	Ischemic stroke	ICD10	Cereb Infrc D/T Unsp Occls Or Stenos Of Unsp Post Cereb Art Or Cerebral Infarction Due To Unspecified Occlusion Or Stenosis Of Unspecified Posterior Cerebral Artery
Diagnosis	I63541	Ischemic stroke	ICD10	Cereb Infrc Due To Unsp Occls Or Stenos Of Right Cereblr Art Or Cerebral Infarction Due To Unspecified Occlusion Or Stenosis Of Right Cerebellar Artery
Diagnosis	I63542	Ischemic stroke	ICD10	Cereb Infrc Due To Unsp Occls Or Stenos Of Left Cereblr Art Or Cerebral Infarction Due To Unspecified Occlusion Or Stenosis Of Left Cerebellar Artery
Diagnosis	I63543	Ischemic stroke	ICD10	Cerebral Infarction Due To Unspecified Occlusion Or Stenosis Of Bilateral Cerebellar Arteries
Diagnosis	I63549	Ischemic stroke	ICD10	Cereb Infrc Due To Unsp Occls Or Stenos Of Unsp Cereblr Art Or Cerebral Infarction Due To Unspecified Occlusion Or Stenosis Of Unspecified Cerebellar Artery
Diagnosis	I6359	Ischemic stroke	ICD10	Cereb Infrc Due To Unsp Occls Or Stenosis Of Cerebral Artery Or Cerebral Infarction Due To Unspecified Occlusion Or Stenosis Of Other Cerebral Artery

Diagnosis	I636	Ischemic stroke	ICD10	Cerebral Infarction Due To Cerebral Venous Thrombosis, Nonpyogenic Or Cerebral Infrc Due To Cerebral Venous Thombos, Nonpyogenic
Diagnosis	I638	Ischemic stroke	ICD10	Other Cerebral Infarction
Diagnosis	I639	Ischemic stroke	ICD10	Cerebral Infarction, Unspecified
Diagnosis	I6789	Ischemic stroke	ICD10	Other Cerebrovascular Disease
Diagnosis	436	Ischemic stroke	ICD9	Cva
Diagnosis	43301	Ischemic stroke	ICD9	Ocl Bslr Art W Infrct
Diagnosis	43311	Ischemic stroke	ICD9	Ocl Crtd Art W Infrct
Diagnosis	43321	Ischemic stroke	ICD9	Ocl Vrtb Art W Infrct
Diagnosis	43331	Ischemic stroke	ICD9	Ocl Mlt Bi Art W Infrct
Diagnosis	43381	Ischemic stroke	ICD9	Ocl Spcf Art W Infrct
Diagnosis	43391	Ischemic stroke	ICD9	Ocl Art Nos W Infrct
Diagnosis	43401	Ischemic stroke	ICD9	Crbl Thrmbs W Infrct
Diagnosis	43411	Ischemic stroke	ICD9	Crbl Emblsm W Infrct
Diagnosis	43491	Ischemic stroke	ICD9	Crbl Art Ocl Nos W Infrc
Diagnosis	4441	Systemic Embolism	9	thoracic aortic embolism
Diagnosis	44401	Systemic Embolism	9	saddle embolus abd aorta
Diagnosis	44489	Systemic Embolism	9	arterial embolism nec
Diagnosis	44421	Systemic Embolism	9	upper extremity embolism
Diagnosis	44502	Systemic Embolism	9	atheroembolism,lower ext
Diagnosis	44481	Systemic Embolism	9	iliac artery embolism
Diagnosis	44589	Systemic Embolism	9	atheroembolism, site nec
Diagnosis	4440	Systemic Embolism	9	abd aortic embolism
Diagnosis	44409	Systemic Embolism	9	ot art emb/thrm abd aort

Diagnosis	44422	Systemic Embolism	9	lower extremity embolism
Diagnosis	44501	Systemic Embolism	9	atheroembolism,upper ext
Diagnosis	44581	Systemic Embolism	9	atheroembolism, kidney
Diagnosis	4449	Systemic Embolism	9	arterial embolism nos
Diagnosis	I745	Systemic Embolism	10	embolism and thrombosis of iliac artery
Diagnosis	I75012	Systemic Embolism	10	atheroembolism of left upper extremity
Diagnosis	I7411	Systemic Embolism	10	embolism and thrombosis of thoracic aorta
Diagnosis	I7401	Systemic Embolism	10	saddle embolus of abdominal aorta
Diagnosis	I7581	Systemic Embolism	10	atheroembolism of kidney
Diagnosis	I75022	Systemic Embolism	10	atheroembolism of left lower extremity
Diagnosis	I748	Systemic Embolism	10	embolism and thrombosis of other arteries
Diagnosis	I7589	Systemic Embolism	10	atheroembolism of other site
Diagnosis	I7409	Systemic Embolism	10	other arterial embolism and thrombosis of abdominal aorta
Diagnosis	I75021	Systemic Embolism	10	atheroembolism of right lower extremity
Diagnosis	I75023	Systemic Embolism	10	atheroembolism of bilateral lower extremities
Diagnosis	I75029	Systemic Embolism	10	atheroembolism of unspecified lower extremity
Diagnosis	I7410	Systemic Embolism	10	embolism and thrombosis of unspecified parts of aorta
Diagnosis	I744	Systemic Embolism	10	embolism and thrombosis of arteries of extremities, unsp or embolism and thrombosis of arteries of extremities, unspecified
Diagnosis	I75013	Systemic Embolism	10	atheroembolism of bilateral upper extremities
Diagnosis	I7419	Systemic Embolism	10	embolism and thrombosis of other parts of aorta
Diagnosis	I75011	Systemic Embolism	10	atheroembolism of right upper extremity
Diagnosis	I75019	Systemic Embolism	10	atheroembolism of unspecified upper extremity
Diagnosis	I743	Systemic Embolism	10	embolism and thrombosis of arteries of the lower extremities

Diagnosis	I749	Systemic Embolism	10	embolism and thrombosis of unspecified artery
Diagnosis	I742	Systemic Embolism	10	embolism and thrombosis of arteries of the upper extremities
Diagnosis	53241	Major Bleeding	9	chr duoden ulc hem-obstr
Diagnosis	53341	Major Bleeding	9	chr peptic ulc w hem-obs
Diagnosis	5693	Major Bleeding	9	rectal & anal hemorrhage
Diagnosis	53160	Major Bleeding	9	chr stomach ulc hem/perf
Diagnosis	53420	Major Bleeding	9	ac margin ulc w hem/perf
Diagnosis	53121	Major Bleeding	9	ac stom ulc hem/perf-obs
Diagnosis	53561	Major Bleeding	9	duodenitis w hmrhg
Diagnosis	56202	Major Bleeding	9	dvrtclo sml int w hmrhg
Diagnosis	53221	Major Bleeding	9	ac duod ulc hem/perf-obs
Diagnosis	53461	Major Bleeding	9	chr marg ulc hem/perf-ob
Diagnosis	56203	Major Bleeding	9	dvrtcli sml int w hmrhg
Diagnosis	53260	Major Bleeding	9	chr duoden ulc hem/perf
Diagnosis	53521	Major Bleeding	9	gstr mcsl hyprt w hmrg
Diagnosis	53401	Major Bleeding	9	ac margin ulc w hem-obst
Diagnosis	53321	Major Bleeding	9	ac pept ulc hem/perf-obs
Diagnosis	53101	Major Bleeding	9	ac stomac ulc w hem-obst
Diagnosis	53200	Major Bleeding	9	ac duodenal ulcer w hem
Diagnosis	53140	Major Bleeding	9	chr stomach ulc w hem
Diagnosis	53531	Major Bleeding	9	alchl gstritis w hmrhg
Diagnosis	53440	Major Bleeding	9	chr marginal ulcer w hem
Diagnosis	53301	Major Bleeding	9	ac peptic ulc w hem-obst

Diagnosis	53261	Major Bleeding	9	chr duod ulc hem/perf-ob
Diagnosis	53240	Major Bleeding	9	chr duoden ulcer w hem
Diagnosis	53441	Major Bleeding	9	chr margin ulc w hem-obs
Diagnosis	53340	Major Bleeding	9	chr peptic ulcer w hem
Diagnosis	5780	Major Bleeding	9	hematemesis
Diagnosis	5781	Major Bleeding	9	blood in stool
Diagnosis	53141	Major Bleeding	9	chr stom ulc w hem-obstr
Diagnosis	53551	Major Bleeding	9	gstr/ddnts nos w hmrhg
Diagnosis	53421	Major Bleeding	9	ac marg ulc hem/perf-obs
Diagnosis	53400	Major Bleeding	9	ac marginal ulcer w hem
Diagnosis	53360	Major Bleeding	9	chr pept ulc w hem/perf
Diagnosis	53460	Major Bleeding	9	chr margin ulc hem/perf
Diagnosis	53082	Major Bleeding	9	esophageal hemorrhage
Diagnosis	53220	Major Bleeding	9	ac duoden ulc w hem/perf
Diagnosis	53501	Major Bleeding	9	acute gastritis w hmrhg
Diagnosis	53783	Major Bleeding	9	angio stm/dudn w hmrhg
Diagnosis	53300	Major Bleeding	9	ac peptic ulcer w hemorr
Diagnosis	4560	Major Bleeding	9	esophag varices w bleed
Diagnosis	53361	Major Bleeding	9	chr pept ulc hem/perf-ob
Diagnosis	5789	Major Bleeding	9	gastrointest hemorr nos
Diagnosis	53320	Major Bleeding	9	ac peptic ulc w hem/perf
Diagnosis	53201	Major Bleeding	9	ac duoden ulc w hem-obst
Diagnosis	45620	Major Bleeding	9	bleed esoph var oth dis

Diagnosis	53511	Major Bleeding	9	atrph gastritis w hmrhg
Diagnosis	53161	Major Bleeding	9	chr stom ulc hem/perf-ob
Diagnosis	56881	Major Bleeding	9	hemoperitoneum
Diagnosis	56213	Major Bleeding	9	dvrtcli colon w hmrhg
Diagnosis	56985	Major Bleeding	9	angio intes w hmrhg
Diagnosis	53120	Major Bleeding	9	ac stomac ulc w hem/perf
Diagnosis	53541	Major Bleeding	9	oth spf gastrt w hmrhg
Diagnosis	56212	Major Bleeding	9	dvrtclo colon w hmrhg
Diagnosis	53100	Major Bleeding	9	ac stomach ulcer w hem
Diagnosis	K5733	Major Bleeding	10	diverticulitis of large intestine without perforation or abscess with bleeding or dvtrcli of lg int w/o perforation or abscess w bleeding
Diagnosis	K91841	Major Bleeding	10	postproc hemor/hemtom of a dgstv sys org fol oth procedure or postprocedural hemorrhage and hematoma of a digestive system organ or structure following other procedure or postprocedural hemorrhage of a digestive system organ or structure following other p
Diagnosis	K661	Major Bleeding	10	hemoperitoneum
Diagnosis	K5731	Major Bleeding	10	diverticulosis of large intestine without perforation or abscess with bleeding or dvrtclos of lg int w/o perforation or abscess w bleeding
Diagnosis	K2981	Major Bleeding	10	duodenitis with bleeding
Diagnosis	K9162	Major Bleeding	10	intraop hemor/hemtom of a dgstv sys org comp oth procedure or intraoperative hemorrhage and hematoma of a digestive system organ or structure complicating other procedure
Diagnosis	K282	Major Bleeding	10	acute gastrojejunal ulcer w both hemorrhage and perforation or acute gastrojejunal ulcer with both hemorrhage and perforation
Diagnosis	K5753	Major Bleeding	10	diverticulitis of both small and large intestine without perforation or abscess with bleeding or dvtrcli of both small and lg int w/o perf or abscess w bleed
Diagnosis	K2961	Major Bleeding	10	other gastritis with bleeding
Diagnosis	K226	Major Bleeding	10	gastro-esophageal laceration-hemorrhage syndrome

Diagnosis	K921	Major Bleeding	10	melena
Diagnosis	K5721	Major Bleeding	10	diverticulitis of large intestine with perforation and abscess with bleeding or dvtrcli of lg int w perforation and abscess w bleeding
Diagnosis	K260	Major Bleeding	10	acute duodenal ulcer with hemorrhage
Diagnosis	K91840	Major Bleeding	10	postproc hemor/hemtom of dgstv sys org fol a dgstv sys proc or postprocedural hemorrhage and hematoma of a digestive system organ or structure following a digestive system procedure or postprocedural hemorrhage of a digestive system organ or structure fol
Diagnosis	K274	Major Bleeding	10	chronic or unsp peptic ulcer, site unsp, with hemorrhage or chronic or unspecified peptic ulcer, site unspecified, with hemorrhage
Diagnosis	K272	Major Bleeding	10	acute peptic ulcer, site unsp, w both hemorrhage and perf or acute peptic ulcer, site unspecified, with both hemorrhage and perforation
Diagnosis	K264	Major Bleeding	10	chronic or unspecified duodenal ulcer with hemorrhage
Diagnosis	K5741	Major Bleeding	10	diverticulitis of both small and large intestine with perforation and abscess with bleeding or dvtrcli of both small and lg int w perf and abscess w bleed
Diagnosis	K266	Major Bleeding	10	chronic or unsp duodenal ulcer w both hemorrhage and perf or chronic or unspecified duodenal ulcer with both hemorrhage and perforation
Diagnosis	K262	Major Bleeding	10	acute duodenal ulcer with both hemorrhage and perforation
Diagnosis	K2931	Major Bleeding	10	chronic superficial gastritis with bleeding
Diagnosis	K2951	Major Bleeding	10	unspecified chronic gastritis with bleeding
Diagnosis	K5701	Major Bleeding	10	diverticulitis of small intestine with perforation and abscess with bleeding or dvtrcli of sm int w perforation and abscess w bleeding
Diagnosis	I8511	Major Bleeding	10	secondary esophageal varices with bleeding
Diagnosis	K5713	Major Bleeding	10	diverticulitis of small intestine without perforation or abscess with bleeding or dvtrcli of sm int w/o perforation or abscess w bleeding
Diagnosis	K286	Major Bleeding	10	chronic or unsp gastrojejunal ulcer w both hemor and perf or chronic or unspecified gastrojejunal ulcer with both hemorrhage and perforation
Diagnosis	K5751	Major Bleeding	10	diverticulosis of both small and large intestine without perforation or abscess with bleeding or dvrtclos of both small and lg int w/o perf or abscs w bleed

Diagnosis	K920	Major Bleeding	10	hematemesis
Diagnosis	K2921	Major Bleeding	10	alcoholic gastritis with bleeding
Diagnosis	K284	Major Bleeding	10	chronic or unspecified gastrojejunal ulcer with hemorrhage
Diagnosis	K5793	Major Bleeding	10	diverticulitis of intestine, part unspecified, without perforation or abscess with bleeding or dvtrcli of intest, part unsp, w/o perf or abscess w bleeding
Diagnosis	K2991	Major Bleeding	10	gastroduodenitis, unspecified, with bleeding
Diagnosis	I8501	Major Bleeding	10	esophageal varices with bleeding
Diagnosis	K252	Major Bleeding	10	acute gastric ulcer with both hemorrhage and perforation
Diagnosis	K9161	Major Bleeding	10	intraop hemor/hemtom of dgstv sys org comp a dgstv sys proc or intraoperative hemorrhage and hematoma of a digestive system organ or structure complicating a digestive system procedure or intraoperative hemorrhage and hematoma of a digestive system organ
Diagnosis	K256	Major Bleeding	10	chronic or unsp gastric ulcer w both hemorrhage and perf or chronic or unspecified gastric ulcer with both hemorrhage and perforation
Diagnosis	K280	Major Bleeding	10	acute gastrojejunal ulcer with hemorrhage
Diagnosis	K5791	Major Bleeding	10	diverticulosis of intestine, part unspecified, without perforation or abscess with bleeding or dvrtclos of intest, part unsp, w/o perf or abscess w bleed
Diagnosis	K922	Major Bleeding	10	gastrointestinal hemorrhage, unspecified
Diagnosis	K2901	Major Bleeding	10	acute gastritis with bleeding
Diagnosis	K2971	Major Bleeding	10	gastritis, unspecified, with bleeding
Diagnosis	K270	Major Bleeding	10	acute peptic ulcer, site unspecified, with hemorrhage
Diagnosis	K3182	Major Bleeding	10	dieulafoy lesion (hemorrhagic) of stomach and duodenum
Diagnosis	K6381	Major Bleeding	10	dieulafoy lesion of intestine
Diagnosis	K2211	Major Bleeding	10	ulcer of esophagus with bleeding
Diagnosis	K254	Major Bleeding	10	chronic or unspecified gastric ulcer with hemorrhage
Diagnosis	K31811	Major Bleeding	10	angiodysplasia of stomach and duodenum with bleeding

Diagnosis	K276	Major Bleeding	10	chr or unsp peptic ulcer, site unsp, w both hemor and perf or chronic or unspecified peptic ulcer, site unspecified, with both hemorrhage and perforation
Diagnosis	K5781	Major Bleeding	10	diverticulitis of intestine, part unspecified, with perforation and abscess with bleeding or dvtrcli of intest, part unsp, w perf and abscess w bleeding
Diagnosis	K2941	Major Bleeding	10	chronic atrophic gastritis with bleeding
Diagnosis	K5521	Major Bleeding	10	angiodysplasia of colon with hemorrhage
Diagnosis	K5711	Major Bleeding	10	diverticulosis of small intestine without perforation or abscess with bleeding or dvrtclos of sm int w/o perforation or abscess w bleeding
Diagnosis	K250	Major Bleeding	10	acute gastric ulcer with hemorrhage
Diagnosis	K625	Major Bleeding	10	hemorrhage of anus and rectum
Diagnosis	85246	Major Bleeding	9	extradural hem-coma nos
Diagnosis	85305	Major Bleeding	9	brain hem nec-deep coma
Diagnosis	85204	Major Bleeding	9	subarach hem-prolng coma
Diagnosis	85200	Major Bleeding	9	traum subarachnoid hem
Diagnosis	85222	Major Bleeding	9	subdural hem-brief coma
Diagnosis	85240	Major Bleeding	9	traumatic extradural hem
Diagnosis	4321	Major Bleeding	9	subdural hemorrhage
Diagnosis	85303	Major Bleeding	9	brain hem nec-mod coma
Diagnosis	85201	Major Bleeding	9	subarachnoid hem-no coma
Diagnosis	85225	Major Bleeding	9	subdural hem-deep coma
Diagnosis	85249	Major Bleeding	9	extadural hem-concuss
Diagnosis	4329	Major Bleeding	9	intracranial hemorr nos
Diagnosis	85209	Major Bleeding	9	subarach hem-concussion
Diagnosis	85306	Major Bleeding	9	brain hem nec-coma nos
Diagnosis	85245	Major Bleeding	9	extradural hem-deep coma

Diagnosis	431	Major Bleeding	9	intracerebral hemorrhage
Diagnosis	85300	Major Bleeding	9	traumatic brain hem nec
Diagnosis	85244	Major Bleeding	9	extradur hem-proln coma
Diagnosis	85309	Major Bleeding	9	brain hem nec-concussion
Diagnosis	85243	Major Bleeding	9	extradural hem-mod coma
Diagnosis	85220	Major Bleeding	9	traumatic subdural hem
Diagnosis	85224	Major Bleeding	9	subdural hem-prolng coma
Diagnosis	85241	Major Bleeding	9	extradural hem w/o coma
Diagnosis	85302	Major Bleeding	9	brain hem nec-brief coma
Diagnosis	85203	Major Bleeding	9	subarach hem-mod coma
Diagnosis	85206	Major Bleeding	9	subarach hem-coma nos
Diagnosis	85304	Major Bleeding	9	brain hem nec-proln coma
Diagnosis	4320	Major Bleeding	9	nontraum extradural hem
Diagnosis	85221	Major Bleeding	9	subdural hem w/o coma
Diagnosis	85242	Major Bleeding	9	extradur hem-brief coma
Diagnosis	85226	Major Bleeding	9	subdural hemorr-coma nos
Diagnosis	430	Major Bleeding	9	subarachnoid hemorrhage
Diagnosis	85202	Major Bleeding	9	subarach hem-brief coma
Diagnosis	85205	Major Bleeding	9	subarach hem-deep coma
Diagnosis	85223	Major Bleeding	9	subdural hemorr-mod coma
Diagnosis	85229	Major Bleeding	9	subdural hem-concussion
Diagnosis	85301	Major Bleeding	9	brain hem nec w/o coma
Diagnosis	S06347A	Major Bleeding	10	traum hemor r cereb w loc w dth d/t brain inj bf consc, init or traumatic hemorrhage of right cerebrum with loss of

				consciousness of any duration with death due to brain injury prior to regaining consciousness, initial encounter
Diagnosis	S06360A	Major Bleeding	10	traum hemor cereb, w/o loss of consciousness, init or traumatic hemorrhage of cerebrum, unspecified, without loss of consciousness, initial encounter
Diagnosis	S06354A	Major Bleeding	10	traum hemor left cerebrum w loc of 6 hours to 24 hours, init or traumatic hemorrhage of left cerebrum with loss of consciousness of 6 hours to 24 hours, initial encounter
Diagnosis	I621	Major Bleeding	10	nontraumatic extradural hemorrhage
Diagnosis	S06356A	Major Bleeding	10	traum hemor l cereb w loc >24 hr w/o ret consc w surv, init or traumatic hemorrhage of left cerebrum with loss of consciousness greater than 24 hours without return to pre-existing conscious level with patient surviving, initial encounter
Diagnosis	S064X5A	Major Bleeding	10	epidural hemorrhage w loc >24 hr w ret consc lev, init or epidural hemorrhage with loss of consciousness greater than 24 hours with return to pre-existing conscious level, initial encounter
Diagnosis	I6001	Major Bleeding	10	nontraumatic subarachnoid hemorrhage from right carotid siphon and bifurcation or ntrm subarach hemor from right carotid siphon and bifurc
Diagnosis	S06346A	Major Bleeding	10	traum hemor r cereb w loc >24 hr w/o ret consc w surv, init or traumatic hemorrhage of right cerebrum with loss of consciousness greater than 24 hours without return to pre-existing conscious level with patient surviving, initial encounter
Diagnosis	I6000	Major Bleeding	10	nontraumatic subarachnoid hemorrhage from unspecified carotid siphon and bifurcation or ntrm subarach hemorrhage from unsp carotid siphon and bifurc
Diagnosis	S06350A	Major Bleeding	10	traum hemor left cerebrum w/o loss of consciousness, init or traumatic hemorrhage of left cerebrum without loss of consciousness, initial encounter
Diagnosis	I6200	Major Bleeding	10	nontraumatic subdural hemorrhage, unspecified
Diagnosis	S06345A	Major Bleeding	10	traum hemor r cereb w loc >24 hr w ret consc lev, init or traumatic hemorrhage of right cerebrum with loss of consciousness greater than 24 hours with return to pre-existing conscious level, initial encounter
Diagnosis	S065X7A	Major Bleeding	10	traum subdr hem w loc w dth d/t brain inj bef reg consc,init or traumatic subdural hemorrhage with loss of consciousness of any duration with death due to brain injury before regaining consciousness, initial encounter
Diagnosis	I608	Major Bleeding	10	other nontraumatic subarachnoid hemorrhage
Diagnosis	I604	Major Bleeding	10	nontraumatic subarachnoid hemorrhage from basilar artery

Diagnosis	I618	Major Bleeding	10	other nontraumatic intracerebral hemorrhage
Diagnosis	S065X0A	Major Bleeding	10	traum subdr hem w/o loss of consciousness, init or traumatic subdural hemorrhage without loss of consciousness, initial encounter
Diagnosis	I6032	Major Bleeding	10	nontraumatic subarachnoid hemorrhage from left posterior communicating artery or ntrm subarach hemor from left posterior communicating artery
Diagnosis	I612	Major Bleeding	10	nontraumatic intracerebral hemorrhage in hemisphere, unsp or nontraumatic intracerebral hemorrhage in hemisphere, unspecified
Diagnosis	S064X3A	Major Bleeding	10	epidural hemorrhage w loc of 1-5 hrs 59 min, init or epidural hemorrhage with loss of consciousness of 1 hour to 5 hours 59 minutes, initial encounter
Diagnosis	I615	Major Bleeding	10	nontraumatic intracerebral hemorrhage, intraventricular
Diagnosis	S06369A	Major Bleeding	10	traum hemor cereb, w loc of unsp duration, init or traumatic hemorrhage of cerebrum, unspecified, with loss of consciousness of unspecified duration, initial encounter
Diagnosis	I6031	Major Bleeding	10	nontraumatic subarachnoid hemorrhage from right posterior communicating artery or ntrm subarach hemor from right post communicating artery
Diagnosis	I606	Major Bleeding	10	nontraumatic subarachnoid hemorrhage from oth intracran art or nontraumatic subarachnoid hemorrhage from other intracranial arteries
Diagnosis	S066X7A	Major Bleeding	10	traum subrac hem w loc w death d/t brain inj bf consc, init or traumatic subarachnoid hemorrhage with loss of consciousness of any duration with death due to brain injury prior to regaining consciousness, initial encounter
Diagnosis	S066X0A	Major Bleeding	10	traum subrac hem w/o loss of consciousness, init or traumatic subarachnoid hemorrhage without loss of consciousness, initial encounter
Diagnosis	S064X9A	Major Bleeding	10	epidural hemorrhage w loc of unsp duration, init or epidural hemorrhage with loss of consciousness of unspecified duration, initial encounter
Diagnosis	S06342A	Major Bleeding	10	traum hemor right cerebrum w loc of 31-59 min, init or traumatic hemorrhage of right cerebrum with loss of consciousness of 31 minutes to 59 minutes, initial encounter
Diagnosis	S064X6A	Major Bleeding	10	epidural hemorrhage w loc >24 hr w/o ret consc w surv, init or epidural hemorrhage with loss of consciousness greater than 24 hours without return to pre-existing conscious level with patient surviving, initial encounter
Diagnosis	S066X1A	Major Bleeding	10	traum subrac hem w loc of 30 minutes or less, init or traumatic subarachnoid hemorrhage with loss of consciousness of 30 minutes or less, initial encounter
Diagnosis	S065X8A	Major Bleeding	10	traum subdr hem w loc w dth d/t oth cause bef reg consc,init or traumatic subdural hemorrhage with loss of consciousness

				of any duration with death due to other cause before regaining consciousness, initial encounter
Diagnosis	S066X6A	Major Bleeding	10	traum subrac hem w loc >24 hr w/o ret consc w surv, init or traumatic subarachnoid hemorrhage with loss of consciousness greater than 24 hours without return to pre-existing conscious level with patient surviving, initial encounter
Diagnosis	S066X8A	Major Bleeding	10	traum subrac hem w loc w death d/t oth cause bf consc, init or traumatic subarachnoid hemorrhage with loss of consciousness of any duration with death due to other cause prior to regaining consciousness, initial encounter
Diagnosis	I6010	Major Bleeding	10	nontraumatic subarachnoid hemorrhage from unspecified middle cerebral artery or ntrm subarach hemorrhage from unsp middle cerebral artery
Diagnosis	S064X4A	Major Bleeding	10	epidural hemorrhage w loc of 6 hours to 24 hours, init or epidural hemorrhage with loss of consciousness of 6 hours to 24 hours, initial encounter
Diagnosis	I607	Major Bleeding	10	nontraumatic subarachnoid hemorrhage from unsp intracran art or nontraumatic subarachnoid hemorrhage from unspecified intracranial artery
Diagnosis	I611	Major Bleeding	10	nontraumatic intcrbl hemorrhage in hemisphere, cortical or nontraumatic intracerebral hemorrhage in hemisphere, cortical
Diagnosis	I613	Major Bleeding	10	nontraumatic intracerebral hemorrhage in brain stem
Diagnosis	I6203	Major Bleeding	10	nontraumatic chronic subdural hemorrhage
Diagnosis	S066X3A	Major Bleeding	10	traum subrac hem w loc of 1-5 hrs 59 min, init or traumatic subarachnoid hemorrhage with loss of consciousness of 1 hour to 5 hours 59 minutes, initial encounter
Diagnosis	S06357A	Major Bleeding	10	traum hemor l cereb w loc w dth d/t brain inj bf consc, init or traumatic hemorrhage of left cerebrum with loss of consciousness of any duration with death due to brain injury prior to regaining consciousness, initial encounter
Diagnosis	S066X9A	Major Bleeding	10	traum subrac hem w loc of unsp duration, init or traumatic subarachnoid hemorrhage with loss of consciousness of unspecified duration, initial encounter
Diagnosis	S06364A	Major Bleeding	10	traum hemor cereb, w loc of 6 hours to 24 hours, init or traumatic hemorrhage of cerebrum, unspecified, with loss of consciousness of 6 hours to 24 hours, initial encounter
Diagnosis	S06344A	Major Bleeding	10	traum hemor right cerebrum w loc of 6-24 hrs, init or traumatic hemorrhage of right cerebrum with loss of consciousness of 6 hours to 24 hours, initial encounter
Diagnosis	I609	Major Bleeding	10	nontraumatic subarachnoid hemorrhage, unspecified
Diagnosis	S064X0A	Major Bleeding	10	epidural hemorrhage w/o loss of consciousness, init encntr or epidural hemorrhage without loss of consciousness, initial encounter

Diagnosis	S065X3A	Major Bleeding	10	traum subdr hem w loc of 1-5 hrs 59 min, init or traumatic subdural hemorrhage with loss of consciousness of 1 hour to 5 hours 59 minutes, initial encounter
Diagnosis	S06340A	Major Bleeding	10	traum hemor right cerebrum w/o loss of consciousness, init or traumatic hemorrhage of right cerebrum without loss of consciousness, initial encounter
Diagnosis	I6202	Major Bleeding	10	nontraumatic subacute subdural hemorrhage
Diagnosis	S06355A	Major Bleeding	10	traum hemor left cerebrum w loc >24 hr w ret consc lev, init or traumatic hemorrhage of left cerebrum with loss of consciousness greater than 24 hours with return to pre-existing conscious level, initial encounter
Diagnosis	I6201	Major Bleeding	10	nontraumatic acute subdural hemorrhage
Diagnosis	S066X5A	Major Bleeding	10	traum subrac hem w loc >24 hr w ret consc lev, init or traumatic subarachnoid hemorrhage with loss of consciousness greater than 24 hours with return to pre-existing conscious level, initial encounter
Diagnosis	S065X9A	Major Bleeding	10	traum subdr hem w loc of unsp duration, init or traumatic subdural hemorrhage with loss of consciousness of unspecified duration, initial encounter
Diagnosis	S06358A	Major Bleeding	10	traum hemor l cereb w loc w dth d/t oth cause bf consc, init or traumatic hemorrhage of left cerebrum with loss of consciousness of any duration with death due to other cause prior to regaining consciousness, initial encounter
Diagnosis	S065X2A	Major Bleeding	10	traum subdr hem w loss of consciousness of 31-59 min, init or traumatic subdural hemorrhage with loss of consciousness of 31 minutes to 59 minutes, initial encounter
Diagnosis	S064X7A	Major Bleeding	10	epidur hemor w loc w death d/t brain injury bf consc, init or epidural hemorrhage with loss of consciousness of any duration with death due to brain injury prior to regaining consciousness, initial encounter
Diagnosis	S064X1A	Major Bleeding	10	epidural hemorrhage w loc of 30 minutes or less, init or epidural hemorrhage with loss of consciousness of 30 minutes or less, initial encounter
Diagnosis	S06362A	Major Bleeding	10	traum hemor cereb, w loc of 31-59 min, init or traumatic hemorrhage of cerebrum, unspecified, with loss of consciousness of 31 minutes to 59 minutes, initial encounter
Diagnosis	S06353A	Major Bleeding	10	traum hemor left cerebrum w loc of 1-5 hrs 59 minutes, init or traumatic hemorrhage of left cerebrum with loss of consciousness of 1 hours to 5 hours 59 minutes, initial encounter
Diagnosis	I614	Major Bleeding	10	nontraumatic intracerebral hemorrhage in cerebellum
Diagnosis	S064X8A	Major Bleeding	10	epidur hemor w loc w death due to oth causes bf consc, init or epidural hemorrhage with loss of consciousness of any

				duration with death due to other causes prior to regaining consciousness, initial encounter
Diagnosis	S06348A	Major Bleeding	10	traum hemor r cereb w loc w dth d/t oth cause bf consc, init or traumatic hemorrhage of right cerebrum with loss of consciousness of any duration with death due to other cause prior to regaining consciousness, initial encounter
Diagnosis	S06351A	Major Bleeding	10	traum hemor left cerebrum w loc of 30 minutes or less, init or traumatic hemorrhage of left cerebrum with loss of consciousness of 30 minutes or less, initial encounter
Diagnosis	S06366A	Major Bleeding	10	traum hemor cereb, w loc >24 hr w/o ret consc w surv, init or traumatic hemorrhage of cerebrum, unspecified, with loss of consciousness greater than 24 hours without return to pre-existing conscious level with patient surviving, initial encounter
Diagnosis	I6052	Major Bleeding	10	nontraumatic subarachnoid hemorrhage from l verteb art or nontraumatic subarachnoid hemorrhage from left vertebral artery
Diagnosis	S06361A	Major Bleeding	10	traum hemor cereb, w loc of 30 minutes or less, init or traumatic hemorrhage of cerebrum, unspecified, with loss of consciousness of 30 minutes or less, initial encounter
Diagnosis	S065X1A	Major Bleeding	10	traum subdr hem w loc of 30 minutes or less, init or traumatic subdural hemorrhage with loss of consciousness of 30 minutes or less, initial encounter
Diagnosis	I610	Major Bleeding	10	nontraumatic intcrbl hemorrhage in hemisphere, subcortical or nontraumatic intracerebral hemorrhage in hemisphere, subcortical
Diagnosis	S06349A	Major Bleeding	10	traum hemor right cerebrum w loc of unsp duration, init or traumatic hemorrhage of right cerebrum with loss of consciousness of unspecified duration, initial encounter
Diagnosis	I6011	Major Bleeding	10	nontraumatic subarachnoid hemorrhage from right middle cerebral artery or ntrm subarach hemorrhage from right middle cerebral artery
Diagnosis	I6030	Major Bleeding	10	nontraumatic subarachnoid hemorrhage from unspecified posterior communicating artery or ntrm subarach hemor from unsp posterior communicating artery
Diagnosis	S06359A	Major Bleeding	10	traum hemor left cerebrum w loc of unsp duration, init or traumatic hemorrhage of left cerebrum with loss of consciousness of unspecified duration, initial encounter
Diagnosis	S065X5A	Major Bleeding	10	traum subdr hem w loc >24 hr w ret consc lev, init or traumatic subdural hemorrhage with loss of consciousness greater than 24 hours with return to pre-existing conscious level, initial encounter
Diagnosis	S06343A	Major Bleeding	10	traum hemor right cerebrum w loc of 1-5 hrs 59 minutes, init or traumatic hemorrhage of right cerebrum with loss of consciousness of 1 hours to 5 hours 59 minutes, initial encounter

Diagnosis	S066X4A	Major Bleeding	10	traum subrac hem w loc of 6 hours to 24 hours, init or traumatic subarachnoid hemorrhage with loss of consciousness of 6 hours to 24 hours, initial encounter
Diagnosis	S066X2A	Major Bleeding	10	traum subrac hem w loss of consciousness of 31-59 min, init or traumatic subarachnoid hemorrhage with loss of consciousness of 31 minutes to 59 minutes, initial encounter
Diagnosis	S06363A	Major Bleeding	10	traum hemor cereb, w loc of 1-5 hrs 59 minutes, init or traumatic hemorrhage of cerebrum, unspecified, with loss of consciousness of 1 hours to 5 hours 59 minutes, initial encounter
Diagnosis	S064X2A	Major Bleeding	10	epidural hemorrhage w loc of 31-59 min, init or epidural hemorrhage with loss of consciousness of 31 minutes to 59 minutes, initial encounter
Diagnosis	S06341A	Major Bleeding	10	traum hemor right cerebrum w loc of 30 minutes or less, init or traumatic hemorrhage of right cerebrum with loss of consciousness of 30 minutes or less, initial encounter
Diagnosis	I6012	Major Bleeding	10	nontraumatic subarachnoid hemorrhage from left middle cerebral artery or ntrm subarach hemorrhage from left middle cerebral artery
Diagnosis	I616	Major Bleeding	10	nontraumatic intracerebral hemorrhage, multiple localized
Diagnosis	S06365A	Major Bleeding	10	traum hemor cereb, w loc >24 hr w ret consc lev, init or traumatic hemorrhage of cerebrum, unspecified, with loss of consciousness greater than 24 hours with return to pre-existing conscious level, initial encounter
Diagnosis	S065X4A	Major Bleeding	10	traum subdr hem w loc of 6 hours to 24 hours, init or traumatic subdural hemorrhage with loss of consciousness of 6 hours to 24 hours, initial encounter
Diagnosis	I6050	Major Bleeding	10	nontraumatic subarachnoid hemorrhage from unsp verteb art or nontraumatic subarachnoid hemorrhage from unspecified vertebral artery
Diagnosis	S06367A	Major Bleeding	10	traum hemor cereb, w loc w dth d/t brain inj bf consc, init or traumatic hemorrhage of cerebrum, unspecified, with loss of consciousness of any duration with death due to brain injury prior to regaining consciousness, initial encounter
Diagnosis	S065X6A	Major Bleeding	10	traum subdr hem w loc >24 hr w/o ret consc w surv, init or traumatic subdural hemorrhage with loss of consciousness greater than 24 hours without return to pre-existing conscious level with patient surviving, initial encounter
Diagnosis	S06368A	Major Bleeding	10	traum hemor cereb, w loc w dth d/t oth cause bf consc, init or traumatic hemorrhage of cerebrum, unspecified, with loss of consciousness of any duration with death due to other cause prior to regaining consciousness, initial encounter
Diagnosis	I629	Major Bleeding	10	nontraumatic intracranial hemorrhage, unspecified
Diagnosis	I602	Major Bleeding	10	nontraumatic subarachnoid hemorrhage from anterior communicating artery

Diagnosis	I619	Major Bleeding	10	nontraumatic intracerebral hemorrhage, unspecified
Diagnosis	I6051	Major Bleeding	10	nontraumatic subarachnoid hemorrhage from r verteb art or nontraumatic subarachnoid hemorrhage from right vertebral artery
Diagnosis	I6002	Major Bleeding	10	nontraumatic subarachnoid hemorrhage from left carotid siphon and bifurcation or ntrm subarach hemorrhage from left carotid siphon and bifurc
Diagnosis	S06352A	Major Bleeding	10	traum hemor left cerebrum w loc of 31-59 min, init or traumatic hemorrhage of left cerebrum with loss of consciousness of 31 minutes to 59 minutes, initial encounter
Diagnosis	71916	Major Bleeding	9	hemarthrosis-l/leg
Diagnosis	78630	Major Bleeding	9	hemoptysis nos
Diagnosis	6262	Major Bleeding	9	excessive menstruation
Diagnosis	6265	Major Bleeding	9	ovulation bleeding
Diagnosis	99702	Major Bleeding	9	iatrogen cv infarc/hmrhg
Diagnosis	37632	Major Bleeding	9	orbital hemorrhage
Diagnosis	78631	Major Bleeding	9	ac idio pul hemrg infant
Diagnosis	36281	Major Bleeding	9	retinal hemorrhage
Diagnosis	36362	Major Bleeding	9	expulsive choroid hemorr
Diagnosis	2851	Major Bleeding	9	ac posthemorrhag anemia
Diagnosis	78639	Major Bleeding	9	hemoptysis nec
Diagnosis	9582	Major Bleeding	9	secondary/recur hemorr
Diagnosis	5967	Major Bleeding	9	bladder wall hemorrhage
Diagnosis	59971	Major Bleeding	9	gross hematuria
Diagnosis	36441	Major Bleeding	9	hyphema
Diagnosis	59970	Major Bleeding	9	hematuria nos
Diagnosis	6268	Major Bleeding	9	menstrual disorder nec
Diagnosis	36243	Major Bleeding	9	hem detach pigmnt epith

Diagnosis	71919	Major Bleeding	9	hemarthrosis-mult jts
Diagnosis	7848	Major Bleeding	9	hemorrhage from throat
Diagnosis	71918	Major Bleeding	9	hemarthrosis-jt nec
Diagnosis	71910	Major Bleeding	9	hemarthrosis-unspec
Diagnosis	71915	Major Bleeding	9	hemarthrosis-pelvis
Diagnosis	6267	Major Bleeding	9	postcoital bleeding
Diagnosis	37481	Major Bleeding	9	hemorrhage of eyelid
Diagnosis	71913	Major Bleeding	9	hemarthrosis-forearm
Diagnosis	71912	Major Bleeding	9	hemarthrosis-up/arm
Diagnosis	59972	Major Bleeding	9	microscopic hematuria
Diagnosis	4230	Major Bleeding	9	hemopericardium
Diagnosis	71914	Major Bleeding	9	hemarthrosis-hand
Diagnosis	7847	Major Bleeding	9	epistaxis
Diagnosis	6269	Major Bleeding	9	menstrual disorder nos
Diagnosis	36372	Major Bleeding	9	hemorr choroid detachmnt
Diagnosis	7827	Major Bleeding	9	spontaneous ecchymoses
Diagnosis	6201	Major Bleeding	9	corpus luteum cyst
Diagnosis	5997	Major Bleeding	9	hematuria
Diagnosis	37742	Major Bleeding	9	optic nerve sheath hemor
Diagnosis	37923	Major Bleeding	9	vitreous hemorrhage
Diagnosis	71917	Major Bleeding	9	hemarthrosis-ankle
Diagnosis	36361	Major Bleeding	9	choroidal hemorrhage nos
Diagnosis	36043	Major Bleeding	9	hemophthalmos

Diagnosis	7863	Major Bleeding	9	hemoptysis
Diagnosis	99811	Major Bleeding	9	hemorrhage complic proc
Diagnosis	37272	Major Bleeding	9	conjunctival hemorrhage
Diagnosis	71911	Major Bleeding	9	hemarthrosis-shlder
Diagnosis	6214	Major Bleeding	9	hematometra
Diagnosis	6021	Major Bleeding	9	prostatic congest/hemorr
Diagnosis	M25062	Major Bleeding	10	hemarthrosis, left knee
Diagnosis	H9521	Major Bleeding	10	intraop hemor/hemtom of ear/mastd comp a proc on ear/mastd or intraoperative hemorrhage and hematoma of ear and mastoid process complicating a procedure on the ear and mastoid process
Diagnosis	M25032	Major Bleeding	10	hemarthrosis, left wrist
Diagnosis	H59313	Major Bleeding	10	postproc hemor/hemtom of eye and adnexa fol an opth proc, bi or postprocedural hemorrhage and hematoma of eye and adnexa following an ophthalmic procedure, bilateral or postprocedural hemorrhage of eye and adnexa following an ophthalmic procedure, bilater
Diagnosis	H3563	Major Bleeding	10	retinal hemorrhage, bilateral
Diagnosis	N938	Major Bleeding	10	other specified abnormal uterine and vaginal bleeding
Diagnosis	G9751	Major Bleeding	10	postproc hemor/hemtom of a nrv sys org fol a nrv sys proc or postprocedural hemorrhage and hematoma of a nervous system organ or structure following a nervous system procedure or postprocedural hemorrhage of a nervous system organ or structure following a
Diagnosis	M96810	Major Bleeding	10	intraop hemor/hemtom of a ms structure comp a ms sys proc or intraoperative hemorrhage and hematoma of a musculoskeletal structure complicating a musculoskeletal system procedure
Diagnosis	H59321	Major Bleeding	10	postproc hemor/hemtom of right eye and adnexa fol oth proc or postprocedural hemorrhage and hematoma of right eye and adnexa following other procedure or postprocedural hemorrhage of right eye and adnexa following other procedure
Diagnosis	E3601	Major Bleeding	10	intraop hemor/hemtom of endo sys org comp an endo sys proc or intraoperative hemorrhage and hematoma of an endocrine system organ or structure complicating an endocrine system procedure

Diagnosis	I97411	Major Bleeding	10	intraop hemor/hemtom of a circ sys org comp card bypass or intraoperative hemorrhage and hematoma of a circulatory system organ or structure complicating a cardiac bypass
Diagnosis	M25052	Major Bleeding	10	hemarthrosis, left hip
Diagnosis	R319	Major Bleeding	10	hematuria, unspecified
Diagnosis	L7601	Major Bleeding	10	intraop hemor/hemtom of skin, subcu comp a dermatologic proc or intraoperative hemorrhage and hematoma of skin and subcutaneous tissue complicating a dermatologic procedure
Diagnosis	M2508	Major Bleeding	10	hemarthrosis, other specified site
Diagnosis	H44819	Major Bleeding	10	hemophthalmos, unspecified eye
Diagnosis	H1133	Major Bleeding	10	conjunctival hemorrhage, bilateral
Diagnosis	L7621	Major Bleeding	10	postproc hemor/hemtom of skin, subcu fol a dermatologic proc or postprocedural hemorrhage and hematoma of skin and subcutaneous tissue following a dermatologic procedure or postprocedural hemorrhage of skin and subcutaneous tissue following a dermatologic
Diagnosis	D62	Major Bleeding	10	acute posthemorrhagic anemia
Diagnosis	M96830	Major Bleeding	10	postproc hemor/hemtom of a ms structure fol a ms sys proc or postprocedural hemorrhage and hematoma of a musculoskeletal structure following a musculoskeletal system procedure or postprocedural hemorrhage of a musculoskeletal structure following a musculo
Diagnosis	H35731	Major Bleeding	10	hemorrhagic detach of retinal pigment epithelium, right eye or hemorrhagic detachment of retinal pigment epithelium, right eye
Diagnosis	D7822	Major Bleeding	10	postproc hemor/hemtom of the spleen following oth procedure or postprocedural hemorrhage and hematoma of the spleen following other procedure or postprocedural hemorrhage of the spleen following other procedure
Diagnosis	H2100	Major Bleeding	10	hyphema, unspecified eye
Diagnosis	L7622	Major Bleeding	10	postproc hemor/hemtom of skin, subcu following oth procedure or postprocedural hemorrhage and hematoma of skin and subcutaneous tissue following other procedure or postprocedural hemorrhage of skin and subcutaneous tissue following other procedure
Diagnosis	J9562	Major Bleeding	10	intraop hemor/hemtom of a resp sys org comp oth procedure or intraoperative hemorrhage and hematoma of a respiratory system organ or structure complicating other procedure
Diagnosis	H05232	Major Bleeding	10	hemorrhage of left orbit

Diagnosis	H3562	Major Bleeding	10	retinal hemorrhage, left eye
Diagnosis	R233	Major Bleeding	10	spontaneous ecchymoses
Diagnosis	D7821	Major Bleeding	10	postprocedural hemor/hemtom of the spleen fol proc on spleen or postprocedural hemorrhage and hematoma of the spleen following a procedure on the spleen or postprocedural hemorrhage of the spleen following a procedure on the spleen
Diagnosis	M25072	Major Bleeding	10	hemarthrosis, left ankle
Diagnosis	L7602	Major Bleeding	10	intraop hemor/hemtom of skin, subcu comp oth procedure or intraoperative hemorrhage and hematoma of skin and subcutaneous tissue complicating other procedure
Diagnosis	H47021	Major Bleeding	10	hemorrhage in optic nerve sheath, right eye
Diagnosis	M25071	Major Bleeding	10	hemarthrosis, right ankle
Diagnosis	H4310	Major Bleeding	10	vitreous hemorrhage, unspecified eye
Diagnosis	H47022	Major Bleeding	10	hemorrhage in optic nerve sheath, left eye
Diagnosis	H35739	Major Bleeding	10	hemorrhagic detach of retinal pigment epithelium, unsp eye or hemorrhagic detachment of retinal pigment epithelium, unspecified eye
Diagnosis	N99820	Major Bleeding	10	postproc hemor/hemtom of a gu sys org fol a gu sys procedure or postprocedural hemorrhage and hematoma of a genitourinary system organ or structure following a genitourinary system procedure or postprocedural hemorrhage of a genitourinary system organ or
Diagnosis	H59121	Major Bleeding	10	intraop hemor/hemtom of right eye and adnexa comp oth proc or intraoperative hemorrhage and hematoma of right eye and adnexa complicating other procedure
Diagnosis	N897	Major Bleeding	10	hematocolpos
Diagnosis	H31303	Major Bleeding	10	unspecified choroidal hemorrhage, bilateral
Diagnosis	M25029	Major Bleeding	10	hemarthrosis, unspecified elbow
Diagnosis	M25076	Major Bleeding	10	hemarthrosis, unspecified foot
Diagnosis	H35732	Major Bleeding	10	hemorrhagic detach of retinal pigment epithelium, left eye or hemorrhagic detachment of retinal pigment epithelium, left eye
Diagnosis	N939	Major Bleeding	10	abnormal uterine and vaginal bleeding, unspecified
Diagnosis	R040	Major Bleeding	10	epistaxis

Diagnosis	H4311	Major Bleeding	10	vitreous hemorrhage, right eye
Diagnosis	H3560	Major Bleeding	10	retinal hemorrhage, unspecified eye
Diagnosis	H59319	Major Bleeding	10	postproc hemor/hemtom of unsp eye and adnx fol an opth proc or postprocedural hemorrhage and hematoma of unspecified eye and adnexa following an ophthalmic procedure or postprocedural hemorrhage of unspecified eye and adnexa following an ophthalmic proced
Diagnosis	H31302	Major Bleeding	10	unspecified choroidal hemorrhage, left eye
Diagnosis	H59113	Major Bleeding	10	intraop hemor/hemtom of eye and adnexa comp an opth proc, bi or intraoperative hemorrhage and hematoma of eye and adnexa complicating an ophthalmic procedure, bilateral
Diagnosis	N421	Major Bleeding	10	congestion and hemorrhage of prostate
Diagnosis	G9752	Major Bleeding	10	postproc hemor/hemtom of a nervous sys org fol oth procedure or postprocedural hemorrhage and hematoma of a nervous system organ or structure following other procedure or postprocedural hemorrhage of a nervous system organ or structure following other pro
Diagnosis	M25075	Major Bleeding	10	hemarthrosis, left foot
Diagnosis	I97610	Major Bleeding	10	postproc hemor/hemtom of a circ sys org fol a cardiac cath or postprocedural hemorrhage and hematoma of a circulatory system organ or structure following a cardiac catheterization or postprocedural hemorrhage of a circulatory system organ or structure fol
Diagnosis	H59312	Major Bleeding	10	postproc hemor/hemtom of l eye and adnexa fol an opth proc or postprocedural hemorrhage and hematoma of left eye and adnexa following an ophthalmic procedure or postprocedural hemorrhage of left eye and adnexa following an ophthalmic procedure
Diagnosis	H59111	Major Bleeding	10	intraop hemor/hemtom of r eye and adnexa comp an opth proc or intraoperative hemorrhage and hematoma of right eye and adnexa complicating an ophthalmic procedure
Diagnosis	E89811	Major Bleeding	10	postproc hemor/hemtom of an endo sys org fol oth procedure or postprocedural hemorrhage and hematoma of an endocrine system organ or structure following other procedure or postprocedural hemorrhage of an endocrine system organ or structure following other
Diagnosis	E89810	Major Bleeding	10	postproc hemor/hemtom of endo sys org fol an endo sys proc or postprocedural hemorrhage and hematoma of an endocrine system organ or structure following an endocrine system procedure or postprocedural hemorrhage of an endocrine system organ or structure f

Diagnosis	M25073	Major Bleeding	10	hemarthrosis, unspecified ankle
Diagnosis	I97410	Major Bleeding	10	intraoperative hemor/hemtom of a circ sys org comp card cath or intraoperative hemorrhage and hematoma of a circulatory system organ or structure complicating a cardiac catheterization
Diagnosis	M25031	Major Bleeding	10	hemarthrosis, right wrist
Diagnosis	J95831	Major Bleeding	10	postproc hemor/hemtom of a resp sys org fol oth procedure or postprocedural hemorrhage and hematoma of a respiratory system organ or structure following other procedure or postprocedural hemorrhage of a respiratory system organ or structure following othe
Diagnosis	I312	Major Bleeding	10	hemopericardium, not elsewhere classified
Diagnosis	M25041	Major Bleeding	10	hemarthrosis, right hand
Diagnosis	H44812	Major Bleeding	10	hemophthalmos, left eye
Diagnosis	G9732	Major Bleeding	10	intraop hemor/hemtom of a nervous sys org comp oth procedure or intraoperative hemorrhage and hematoma of a nervous system organ or structure complicating other procedure
Diagnosis	M25039	Major Bleeding	10	hemarthrosis, unspecified wrist
Diagnosis	H2101	Major Bleeding	10	hyphema, right eye
Diagnosis	H59119	Major Bleeding	10	intraop hemor/hemtom of unsp eye and adnx comp an opth proc or intraoperative hemorrhage and hematoma of unspecified eye and adnexa complicating an ophthalmic procedure
Diagnosis	H9541	Major Bleeding	10	postproc hemor/hemtom of ear/mastd fol proc on ear/mastd or postprocedural hemorrhage and hematoma of ear and mastoid process following a procedure on the ear and mastoid process or postprocedural hemorrhage of ear and mastoid process following a procedur
Diagnosis	H31413	Major Bleeding	10	hemorrhagic choroidal detachment, bilateral
Diagnosis	H31319	Major Bleeding	10	expulsive choroidal hemorrhage, unspecified eye
Diagnosis	M25049	Major Bleeding	10	hemarthrosis, unspecified hand
Diagnosis	M25022	Major Bleeding	10	hemarthrosis, left elbow
Diagnosis	H9522	Major Bleeding	10	intraop hemor/hemtom of ear/mastd complicating oth procedure or intraoperative hemorrhage and hematoma of ear and mastoid process complicating other procedure

Diagnosis	H05233	Major Bleeding	10	hemorrhage of bilateral orbit
Diagnosis	M25051	Major Bleeding	10	hemarthrosis, right hip
Diagnosis	H1132	Major Bleeding	10	conjunctival hemorrhage, left eye
Diagnosis	H1131	Major Bleeding	10	conjunctival hemorrhage, right eye
Diagnosis	H31311	Major Bleeding	10	expulsive choroidal hemorrhage, right eye
Diagnosis	H44811	Major Bleeding	10	hemophthalmos, right eye
Diagnosis	H47029	Major Bleeding	10	hemorrhage in optic nerve sheath, unspecified eye
Diagnosis	H9542	Major Bleeding	10	postproc hemor/hemtom of ear/mastd following oth procedure or postprocedural hemorrhage and hematoma of ear and mastoid process following other procedure or postprocedural hemorrhage of ear and mastoid process following other procedure
Diagnosis	H2102	Major Bleeding	10	hyphema, left eye
Diagnosis	I9742	Major Bleeding	10	intraop hemor/hemtom of a circ sys org comp oth procedure or intraoperative hemorrhage and hematoma of a circulatory system organ or structure complicating other procedure
Diagnosis	H31313	Major Bleeding	10	expulsive choroidal hemorrhage, bilateral
Diagnosis	G9731	Major Bleeding	10	intraop hemor/hemtom of a nervous sys org comp nrv sys proc or intraoperative hemorrhage and hematoma of a nervous system organ or structure complicating a nervous system procedure
Diagnosis	M25011	Major Bleeding	10	hemarthrosis, right shoulder
Diagnosis	M25021	Major Bleeding	10	hemarthrosis, right elbow
Diagnosis	H59123	Major Bleeding	10	intraop hemor/hemtom of eye and adnexa comp oth proc, bi or intraoperative hemorrhage and hematoma of eye and adnexa complicating other procedure, bilateral
Diagnosis	H31301	Major Bleeding	10	unspecified choroidal hemorrhage, right eye
Diagnosis	H59329	Major Bleeding	10	postproc hemor/hemtom of unsp eye and adnexa fol oth proc or postprocedural hemorrhage and hematoma of unspecified eye and adnexa following other procedure or postprocedural hemorrhage of unspecified eye and adnexa following other procedure
Diagnosis	H59129	Major Bleeding	10	intraop hemor/hemtom of unsp eye and adnexa comp oth proc or intraoperative hemorrhage and hematoma of unspecified eye and adnexa complicating other procedure

Diagnosis	H59122	Major Bleeding	10	intraop hemor/hemtom of left eye and adnexa comp oth proc or intraoperative hemorrhage and hematoma of left eye and adnexa complicating other procedure
Diagnosis	H4312	Major Bleeding	10	vitreous hemorrhage, left eye
Diagnosis	T792XXA	Major Bleeding	10	traumatic secondary and recurrent hemor and seroma, init or traumatic secondary and recurrent hemorrhage and seroma, initial encounter
Diagnosis	R049	Major Bleeding	10	hemorrhage from respiratory passages, unspecified
Diagnosis	M25074	Major Bleeding	10	hemarthrosis, right foot
Diagnosis	M2500	Major Bleeding	10	hemarthrosis, unspecified joint
Diagnosis	E3602	Major Bleeding	10	intraop hemor/hemtom of an endo sys org comp oth procedure or intraoperative hemorrhage and hematoma of an endocrine system organ or structure complicating other procedure
Diagnosis	M96811	Major Bleeding	10	intraop hemor/hemtom of a ms structure comp oth procedure or intraoperative hemorrhage and hematoma of a musculoskeletal structure complicating other procedure
Diagnosis	M25042	Major Bleeding	10	hemarthrosis, left hand
Diagnosis	I97620	Major Bleeding	10	postprocedural hemorrhage of a circulatory system organ or structure following other procedure
Diagnosis	R0489	Major Bleeding	10	hemorrhage from other sites in respiratory passages
Diagnosis	M96831	Major Bleeding	10	postproc hemor/hemtom of a ms structure fol oth procedure or postprocedural hemorrhage and hematoma of a musculoskeletal structure following other procedure or postprocedural hemorrhage of a musculoskeletal structure following other procedure
Diagnosis	I97618	Major Bleeding	10	postproc hemor/hemtom of circ sys org fol oth circ sys proc or postprocedural hemorrhage and hematoma of a circulatory system organ or structure following other circulatory system procedure or postprocedural hemorrhage of a circulatory system organ or str
Diagnosis	R58	Major Bleeding	10	hemorrhage, not elsewhere classified
Diagnosis	N9962	Major Bleeding	10	intraop hemor/hemtom of a gu sys org comp oth procedure or intraoperative hemorrhage and hematoma of a genitourinary system organ or structure complicating other procedure
Diagnosis	M25061	Major Bleeding	10	hemarthrosis, right knee
Diagnosis	D7801	Major Bleeding	10	intraop hemor/hemtom of the spleen comp a proc on the spleen or intraoperative hemorrhage and hematoma of the spleen complicating a procedure on the spleen

Diagnosis	N9961	Major Bleeding	10	intraop hemor/hemtom of a gu sys org comp a gu sys procedure or intraoperative hemorrhage and hematoma of a genitourinary system organ or structure complicating a genitourinary system procedure
Diagnosis	H59322	Major Bleeding	10	postproc hemor/hemtom of left eye and adnexa fol oth proc or postprocedural hemorrhage and hematoma of left eye and adnexa following other procedure or postprocedural hemorrhage of left eye and adnexa following other procedure
Diagnosis	H31412	Major Bleeding	10	hemorrhagic choroidal detachment, left eye
Diagnosis	H05239	Major Bleeding	10	hemorrhage of unspecified orbit
Diagnosis	H59311	Major Bleeding	10	postproc hemor/hemtom of r eye and adnexa fol an opth proc or postprocedural hemorrhage and hematoma of right eye and adnexa following an ophthalmic procedure or postprocedural hemorrhage of right eye and adnexa following an ophthalmic procedure
Diagnosis	N857	Major Bleeding	10	hematometra
Diagnosis	H1130	Major Bleeding	10	conjunctival hemorrhage, unspecified eye
Diagnosis	H2103	Major Bleeding	10	hyphema, bilateral
Diagnosis	H31312	Major Bleeding	10	expulsive choroidal hemorrhage, left eye
Diagnosis	H05231	Major Bleeding	10	hemorrhage of right orbit
Diagnosis	N920	Major Bleeding	10	excessive and frequent menstruation with regular cycle
Diagnosis	R310	Major Bleeding	10	gross hematuria
Diagnosis	M25059	Major Bleeding	10	hemarthrosis, unspecified hip
Diagnosis	N930	Major Bleeding	10	postcoital and contact bleeding
Diagnosis	J95830	Major Bleeding	10	postproc hemor/hemtom of a resp sys org fol a resp sys proc or postprocedural hemorrhage and hematoma of a respiratory system organ or structure following a respiratory system procedure or postprocedural hemorrhage of a respiratory system organ or structu
Diagnosis	H31419	Major Bleeding	10	hemorrhagic choroidal detachment, unspecified eye
Diagnosis	J9561	Major Bleeding	10	intraop hemor/hemtom of a resp sys org comp resp sys proc or intraoperative hemorrhage and hematoma of a respiratory system organ or structure complicating a respiratory system procedure

Diagnosis	H47023	Major Bleeding	10	hemorrhage in optic nerve sheath, bilateral
Diagnosis	H4313	Major Bleeding	10	vitreous hemorrhage, bilateral
Diagnosis	N923	Major Bleeding	10	ovulation bleeding
Diagnosis	I97611	Major Bleeding	10	postproc hemor/hemtom of a circ sys org fol cardiac bypass or postprocedural hemorrhage and hematoma of a circulatory system organ or structure following cardiac bypass or postprocedural hemorrhage of a circulatory system organ or structure following card
Diagnosis	H31411	Major Bleeding	10	hemorrhagic choroidal detachment, right eye
Diagnosis	M25019	Major Bleeding	10	hemarthrosis, unspecified shoulder
Diagnosis	I97418	Major Bleeding	10	intraop hemor/hemtom of circ sys org comp oth circ sys proc or intraoperative hemorrhage and hematoma of a circulatory system organ or structure complicating other circulatory system procedure
Diagnosis	H31309	Major Bleeding	10	unspecified choroidal hemorrhage, unspecified eye
Diagnosis	M25012	Major Bleeding	10	hemarthrosis, left shoulder
Diagnosis	R041	Major Bleeding	10	hemorrhage from throat
Diagnosis	H44813	Major Bleeding	10	hemophthalmos, bilateral
Diagnosis	N99821	Major Bleeding	10	postproc hemor/hemtom of a gu sys org fol oth procedure or postprocedural hemorrhage and hematoma of a genitourinary system organ or structure following other procedure or postprocedural hemorrhage of a genitourinary system organ or structure following ot
Diagnosis	D7802	Major Bleeding	10	intraop hemor/hemtom of the spleen comp oth procedure or intraoperative hemorrhage and hematoma of the spleen complicating other procedure
Diagnosis	R042	Major Bleeding	10	hemoptysis
Diagnosis	H3561	Major Bleeding	10	retinal hemorrhage, right eye
Diagnosis	M25069	Major Bleeding	10	hemarthrosis, unspecified knee
Diagnosis	H59323	Major Bleeding	10	postproc hemor/hemtom of eye and adnexa fol oth proc, bi or postprocedural hemorrhage and hematoma of eye and adnexa following other procedure, bilateral or postprocedural hemorrhage of eye and adnexa following other procedure, bilateral

Diagnosis	H35733	Major Bleeding	10	hemorrhagic detach of retinal pigment epithelium, bilateral or hemorrhagic detachment of retinal pigment epithelium, bilateral
Diagnosis	H59112	Major Bleeding	10	intraop hemor/hemtom of l eye and adnexa comp an opth proc or intraoperative hemorrhage and hematoma of left eye and adnexa complicating an ophthalmic procedure
Procedure	4443	Major Bleeding	9	Endoscopic control of gastric or duodenal bleeding
Procedure	9904	Major Bleeding	9	Transfusion of packed cells
Procedure	30230N1	Major Bleeding	10	Transfusion of Nonautologous Red Blood Cells into Peripheral Vein, Open Approach
Procedure	30230P1	Major Bleeding	10	Transfusion of Nonautologous Frozen Red Cells into Peripheral Vein, Open Approach
Procedure	30233N1	Major Bleeding	10	Transfusion of Nonautologous Red Blood Cells into Peripheral Vein, Percutaneous Approach
Procedure	30233P1	Major Bleeding	10	Transfusion of Nonautologous Frozen Red Cells into Peripheral Vein, Percutaneous Approach
Procedure	30240N1	Major Bleeding	10	Transfusion of Nonautologous Red Blood Cells into Central Vein, Open Approach
Procedure	30240P1	Major Bleeding	10	Tansfusion of Nonautologous Frozen Red Cells into Central Vein, Open Approach
Procedure	30243N1	Major Bleeding	10	Transfusion of Nonautologous Red Blood Cells into Central Vein, Percutaneous Approach
Procedure	30243P1	Major Bleeding	10	Transfusion of Nonautologous Frozen Red Cells into Central Vein, Percutaneous Approach
Procedure	30250N1	Major Bleeding	10	Transfusion of Nonautologous Red Blood Cells into Peripheral Artery, Open Approach
Procedure	30250P1	Major Bleeding	10	Transfusion of Nonautologous Frozen Red Cells into Peripheral Artery, Open Approach
Procedure	30253N1	Major Bleeding	10	Transfusion of Nonautologous Red Blood Cells into Peripheral Artery, Percutaneous Approach
Procedure	30253P1	Major Bleeding	10	Transfusion of Nonautologous Frozen Red Cells into Peripheral Artery, Percutaneous Approach
Procedure	30260N1	Major Bleeding	10	Transfusion of Nonautologous Red Blood Cells into Central Artery, Open Approach
Procedure	30260P1	Major Bleeding	10	Transfusion of Nonautologous Frozen Red Cells into Central Artery, Open Approach
Procedure	30263N1	Major Bleeding	10	Transfusion of Nonautologous Red Blood Cells into Central Artery, Percutaneous Approach
Procedure	30263P1	Major Bleeding	10	Transfusion of Nonautologous Frozen Red Cells into Central Artery, Percutaneous Approach
